# Circular RNA CircCDKN2B−AS_006 Promotes the Tumor-like Growth and Metastasis of Rheumatoid Arthritis Synovial Fibroblasts by Targeting the miR−1258/RUNX1 Axis

**DOI:** 10.3390/ijms24065880

**Published:** 2023-03-20

**Authors:** Yayun Xu, Zhuoyan Zai, Zheng Lu, Tao Zhang, Longfei Wang, Xuewen Qian, Jingjing Tao, Xiaoqing Peng, Yihao Zhang, Feihu Chen

**Affiliations:** 1 Department of Epidemiology and Biostatistics, School of Public Health, Anhui Medical University, Hefei 230032, China; 2 Inflammation and Immune Mediated Diseases Laboratory of Anhui Province, Anhui Institute of Innovative Drugs, School of Pharmacy, Anhui Medical University, Hefei 230032, China; 3 The Key Laboratory of Anti-Inflammatory and Immune Medicines, Ministry of Education, Hefei 230032, China; 4 Department of Toxicology, School of Public Health, Anhui Medical University, Hefei 230032, China; 5 Key Laboratory of Environmental Toxicology of Anhui Higher Education Institutes, Hefei 230032, China

**Keywords:** rheumatoid arthritis, rynovial fibroblast, circCDKN2B−AS_006

## Abstract

Rheumatoid arthritis (RA) is an autoimmune polyarthritis in which synovial fibroblasts (SFs) play a major role in cartilage and bone destruction through tumor−like proliferation, migration, and invasion. Circular RNAs (circRNAs) have emerged as vital regulators for tumor progression. However, the regulatory role, clinical significance, and underlying mechanisms of circRNAs in RASF tumor−like growth and metastasis remain largely unknown. Differentially expressed circRNAs in synovium samples from patients with RA and patients with joint trauma were identified via RNA sequencing. Subsequently, in vitro and in vivo experiments were performed to investigate the functional roles of circCDKN2B−AS_006 in RASF proliferation, migration, and invasion. CircCDKN2B−AS_006 was upregulated in synovium samples from patients with RA and promoted the tumor-like proliferation, migration, and invasion of RASFs. Mechanistically, circCDKN2B−AS_006 was shown to regulate the expression of runt−related transcription factor 1 (RUNX1) by sponging miR-1258, influencing the Wnt/β−catenin signaling pathway, and promoting the epithelial−to−mesenchymal transition (EMT) in RASFs. Moreover, in the collagen−induced arthritis (CIA) mouse model, intra−articular injection of lentivirus−shcircCDKN2B−AS_006 was capable of alleviating the severity of arthritis and inhibiting the aggressive behaviors of SFs. Furthermore, the correlation analysis results revealed that the circCDKN2B−AS_006/miR−1258/RUNX1 axis in the synovium was correlated with the clinical indicators of RA patients. CircCDKN2B−AS_006 promoted the proliferation, migration, and invasion of RASFs by modulating the miR−1258/RUNX1 axis.

## 1. Introduction

Rheumatoid arthritis (RA), a common systemic inflammatory autoimmune disease, is characterized by an inflammatory synovium and joint fluid, synovial hyperplasia, and the progressive degradation of cartilage and bone [1]. Epidemiological data have shown that RA affects an estimated 1% of the world’s adult population [2]. The pathogenesis of RA infection remains poorly understood and involves a complex interplay between host factors (such as genetic susceptibilities and aberrant immune responses) and environmental triggers (such as bacterial or viral infection) [3]. Current medical treatments for RA include nonsteroidal anti-inflammatory drugs (NSAIDs), glucocorticoids, nonbiologic disease-modifying antirheumatic drugs (DMARDs), and biologic DMARDs [4]. Despite all these efforts to provide medical therapy for RA, a significant portion of RA patients remain unremitted despite the progression of treatment [5,6]. Therefore, it is crucial to understand the cellular and molecular processes by which the pathology is mediated in order to develop mechanism-driven therapeutic approaches for RA.

Synovial fibroblasts (SFs) in the synovial intimal lining of the joint play a critical role in the pathogenesis of RA through aggressive proliferation and invasion, the secretion of pro-inflammatory cytokines, the formation of pannus, and the production of cartilage-degrading matrix metalloproteases (MMPs) [7,8]. More importantly, activated rheumatoid arthritis synovial fibroblasts (RASFs) exhibit tumor-like features, including an increased proliferation rate and pro-migratory and pro-invasive properties that contribute greatly to pannus formation and joint destruction [9]. Thus, inhibiting the tumor-like features of RASFs may represent potential therapeutic strategies to alleviate RA. Nevertheless, the molecular signatures and biological networks that define the distinct pathophysiologic contributions of SFs in RA have not been systematically clarified.

Circular RNAs (circRNAs), a class of endogenous noncoding RNAs (ncRNAs), are covalently closed−loop molecules without 5′ caps or 3′ poly−A tails, formed via the back−splicing of pre−mRNA [10]. Compared with linear RNAs, circRNAs exhibit higher stability and are more resistant to RNase R digestion [11]. Accumulating evidence has demonstrated that circRNAs can competitively bind to microRNA (miRNA) and act as a “miRNA sponge” to release miRNA’s inhibitory effect on its target genes [12]. Due to their high stability, high conservation, and tissue−specificity, circRNAs have been reported to be implicated in the pathogenesis of various diseases, especially in cancer, and correlated with tumor initiation, progression, and metastasis [13,14]. However, the role and underlying mechanisms of circRNAs in the proliferation, migration, and invasion of activated RASFs in RA remain elusive.

In the present study, we used whole−transcriptome sequencing to screen the differentially expressed circRNAs in the synovia from patients with RA and patients with joint trauma and identified the upregulation of hsa_circCDKN2B−AS_006. We demonstrated that circCDKN2B−AS_006 was notably upregulated in the synovium samples from patients with RA and RASFs. Functionally, circCDKN2B−AS_006 promoted the proliferation, migration, and invasion of RASFs, and circCDKN2B−AS_006 knockdown alleviated arthritis severity in a collagen−induced arthritis (CIA) mouse model. Mechanistically, circCDKN2B−AS_006 was shown to regulate the expression of runt-related transcription factor 1 (RUNX1) by sponging miR−1258, ultimately influencing the Wnt/β−catenin signaling pathway and promoting epithelial−to−mesenchymal transition (EMT) in RASFs. Clinically, circCDKN2B−AS_006, miR−1258, and RUNX1 dysregulation were shown to be related to the clinical indicators of patients with RA. Collectively, these results revealed that the circCDKN2B−AS_006/miR−1258/RUNX1 axis plays a crucial role in RASF proliferation and metastasis and, in particular, enabled us to identify circCDKN2B−AS_006 as a potential biomarker and therapeutic target in RA.

## 2. Results

### 2.1. The Identification and Characteristics of circCDKN2B−AS_006

**Figure 1:**
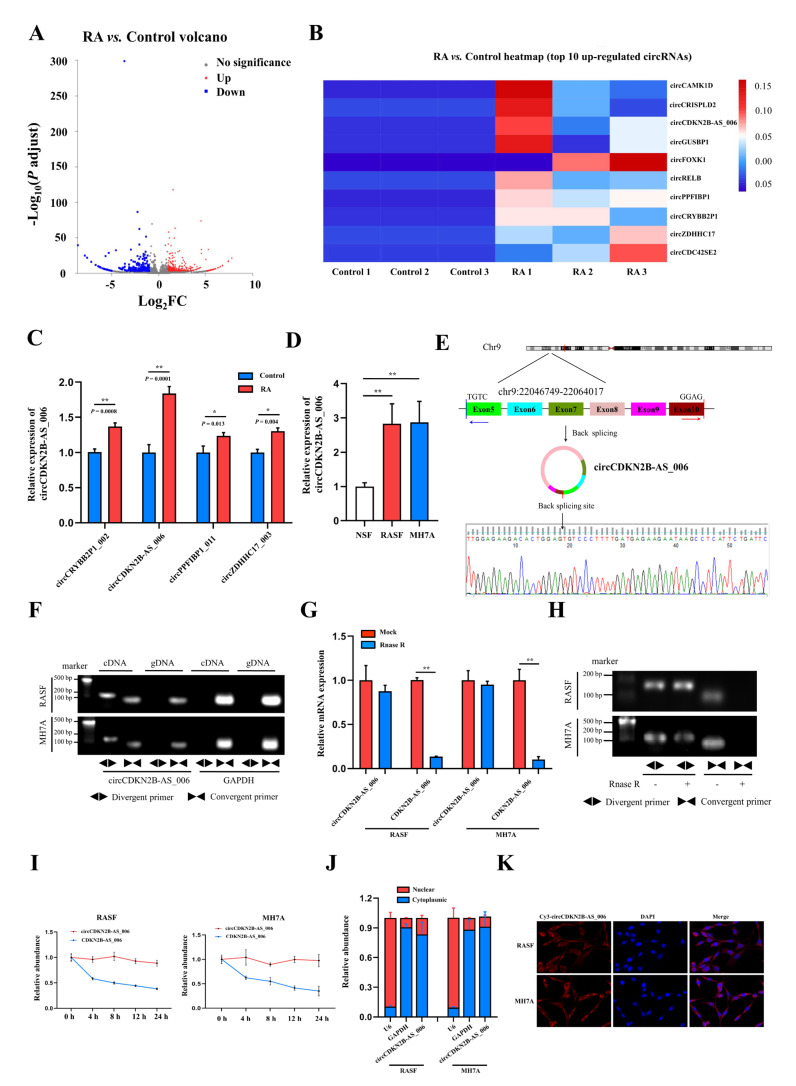
Identification and characterization of circCDKN2B−AS_006. (**A**) Volcano plots showing 283 upregulated and 256 downregulated circRNAs in the synovia from RA patients relative to patients with joint trauma. (**B**) The top 10 upregulated circRNAs. (**C**) Relative circCRYBB2P1_002, circCDKN2B−AS_006, circPPFIBP1_011, and circZDHHC17_003 expressions in the synovia from RA patients (*n* = 40) and patients with joint trauma (*n* = 16) were detected by RT−qPCR. (**D**) Relative circCDKN2B−AS_006 expression in RASFs, MH7A cells, and NSFs was determined via RT−qPCR. (**E**) The schematic illustration showed the back−splicing of circCDKN2B−AS_006, and Sanger sequencing validated the splicing site. (**F**) RT−PCR with divergent and convergent primers and agarose gel electrophoresis analysis were performed to detect the presence of circCDKN2B−AS_006 and its maternal gene *CDKN2B−AS_006* in cDNA and gDNA samples from RASFs and MH7A cells. (**G**,**H**) CircCDKN2B−AS_006 and linear CDKN2B−AS_006 expression in RASFs and MH7A cells were detected by means of RT−qPCR (**G**) and RT−PCR (**H**) after treatment with RNase R or mocks. (**I**) RT−qPCR analysis of the circCDKN2B−AS_006 and CDKN2B−AS_006 expression in RASFs and MH7A cells under treatment with actinomycin D. (**J**) CirCDKN2B−AS_006 was predominantly localized to the cytoplasm of RASFs and MH7A cells, as indicated by a nuclear−cytoplasmic fractionation assay. GAPDH and U6 functioned as normalizing cytoplasmic and nuclear controls, respectively. (**K**) Representative FISH images showing the cellular localization of circCDKN2B−AS_006. ** *p* < 0.01, * *p* < 0.05.

To identify critical circRNAs that contribute to RA progression, we first performed whole−transcriptome sequencing of the synovia from three pairs of patients with RA and patients with joint trauma. A total of 539 differentially expressed circRNAs with a cut−off criteria of fold change > 2.0 and *p* < 0.05 were found, including 283 upregulated circRNAs and 256 downregulated circRNAs (Figure 1A). The top 10 upregulated circRNAs ranked based on fold change were selected for further analysis (Figure 1B). We designed specific primers targeting the junction site of these 10 upregulated circRNAs, and only four circRNAs (circCRYBB2P1_002, circCDKN2B−AS_006, circPPFIBP1_011, and circZDHHC17_003) could be successfully amplified via RT−qPCR. Then, we examined and compared the expression of these four circRNAs via RT−qPCR in the synovia from 40 RA patients and 16 patients with joint trauma, and the difference in the expression of these four circRNAs was statistically significant (Figure 1C). As the *p* value in the comparison of circCDKN2B−AS_006 was the most significant, circCDKN2B−AS_006 was eventually selected for subsequent studies. Consistent with these findings, circCDKN2B−AS_006 was also significantly upregulated in RASFs and MH7A cells (a human rheumatoid arthritis synovial cell line) compared with NSFs (Figure 1D). Sanger sequencing covering the back−splicing junction sequence was performed using the RT−qPCR product of circCDKN2B−AS_006 (Figure 1E). To confirm that circCDKN2B−AS_006 exhibited the characteristic stability of circRNAs, divergent and convergent primers were designed to amplify the circular and linear forms of CDKN2B−AS_006, respectively. Agarose gel electrophoresis analysis of RT−qPCR products indicated that circCDKN2B−AS_006 was amplified only from cDNA, excluding the possibility of genome rearrangement and trans−splicing (Figure 1F). Moreover, after treatment with RNase R (Figure 1G,H) and actinomycin D (Figure 1I), circCDKN2B−AS_006 was more stable than linear CDKN2B−AS_006. In addition, the results of nuclear−cytoplasmic fractionation revealed that circCDKN2B−AS_006 was predominantly localized in the cytoplasm (Figure 1J), which was consistent with the results of the RNA FISH assay (Figure 1K). Collectively, these results identified that circCDKN2B−AS_006 was overexpressed in RASFs and MH7A cells and was mainly localized in the cytoplasm as a highly stable circRNA.

### 2.2. CircCDKN2B−AS_006 Promotes the Proliferation, Migration, and Invasion of RASFs and MH7A Cells In Vitro

**Figure 2:**
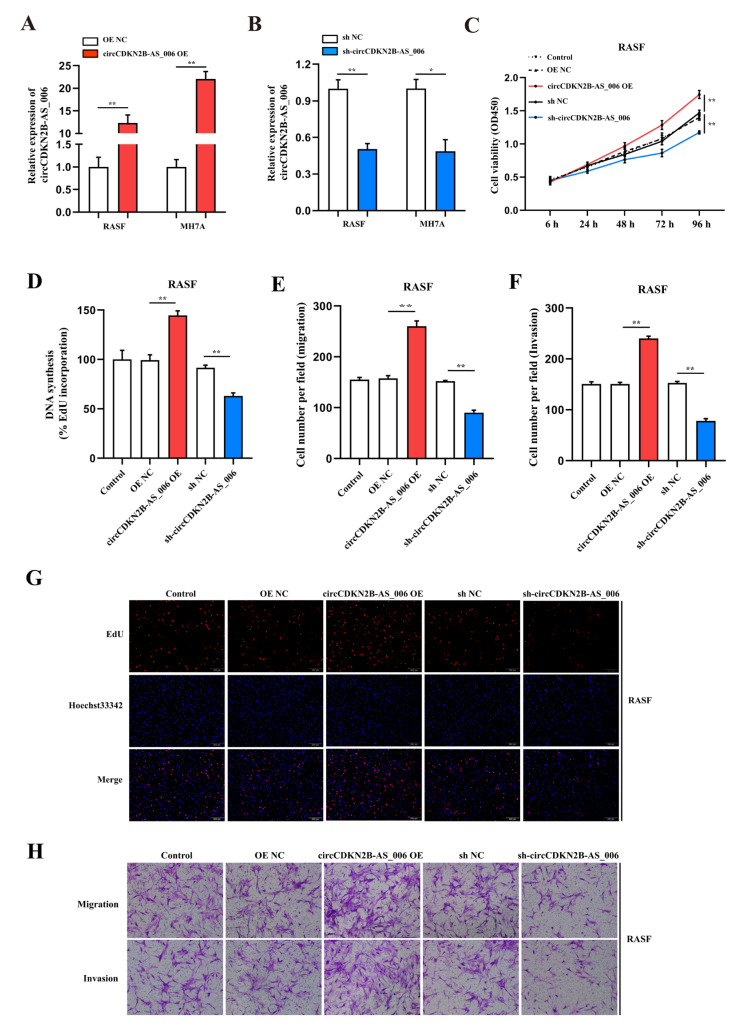
CircCDKN2B−AS_006 promotes the proliferation, migration, and invasion of RASFs in vitro. (**A**,**B**) The efficiency of circCDKN2B−AS_006 overexpression (**A**) and silencing (**B**) in RASFs and MH7A cells was verified by means of RT−qPCR. (**C**) The growth curves of RASFs with circCDKN2B−AS_006 knockdown and overexpression were monitored via CCK−8 assays. (**D**,**G**) EdU assays show that knockdown of circCDKN2B−AS_006 inhibited the DNA synthesis of RASFs, whereas the overexpression of circCDKN2B−AS_006 promoted DNA synthesis in RASFs. (**E**,**F**,**H**) The cell migration and invasion abilities of RASFs were assessed via transwell assays. The samples were imaged at 100 × magnification. ** *p* < 0.01, * *p* < 0.05.

To assess the proliferation and aggressiveness of circCDKN2B−AS_006 in RASFs and MH7A cells, gain− and loss−of−function assays were conducted. RASFs and MH7A cells were transfected with the circCDKN2B−AS_006 plasmid to overexpress circCDKN2B−AS_006. An shRNA targeting the back-splice site of circCDKN2B−AS_006 was constructed to specifically downregulate the expression of circCDKN2B−AS_006 in RASFs and MH7A cells. The efficiency and specificity of circCDKN2B−AS_006 overexpression (Figure 2A) and knockdown (Figure 2B) in RASFs and MH7A cells were verified by means of RT−qPCR. CCK−8 and EdU assays showed that the overexpression of circCDKN2B−AS_006 increased the proliferation of RASFs (Figure 2C,D,G) and MH7A cells (Supplementary Figure S1A,B,E), whereas circCDKN2B−AS_006 downregulation decreased cell growth. In the transwell assay, we found that circCDKN2B−AS_006 overexpression increased the migration and invasion of RASFs (Figure 2E,F,H) and MH7A cells (Supplementary Figure S1C,D,F), whereas circCDKN2B−AS_006 knockdown had the opposite effect. These results indicate that circCDKN2B−AS_006 facilitates the proliferation, migration, and invasion of RASFs and MH7A cells in vitro.

### 2.3. CircCDKN2B−AS_006 Serves as a Sponge for miR-1258

**Figure 3:**
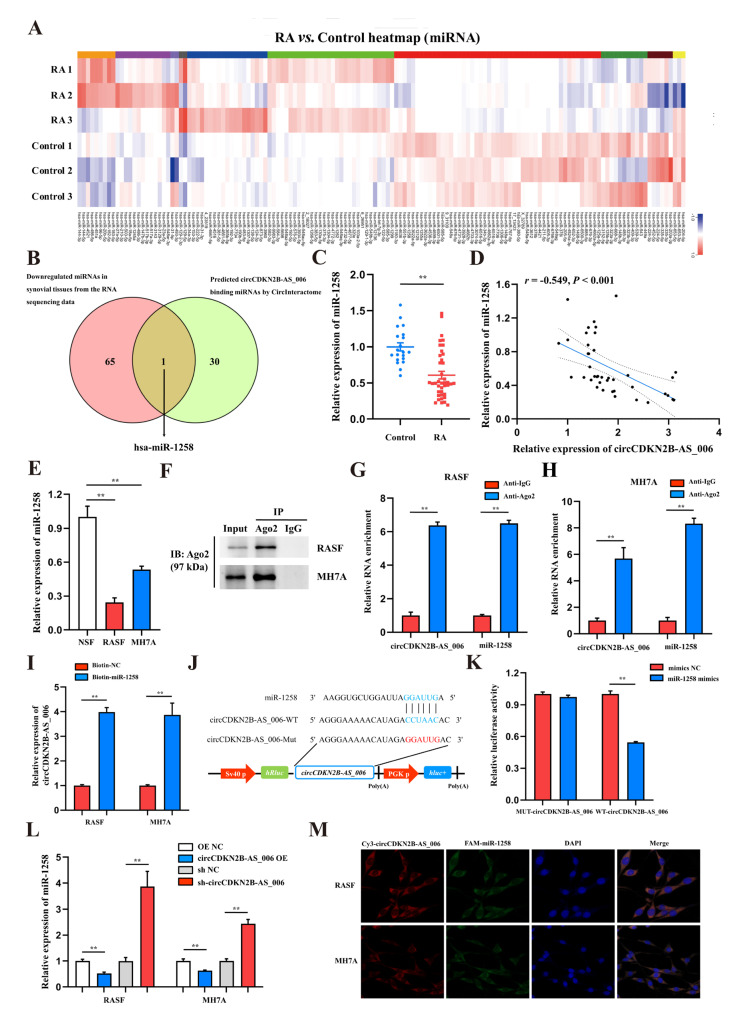
CircCDKN2B−AS_006 serves as a sponge for miR−1258. (**A**) Heatmap of differentially expressed miRNAs in two groups. (**B**) Venn diagram showing the overlapping of the target miRNAs of circCDKN2B−AS_006 predicted based on the circular RNA interactome and the results of whole−transcriptome sequencing (downregulated miRNAs). (**C**) The relative expression of miR−1258 was detected in the synovia from RA patients (*n* = 40) and non−RA patients (*n* = 16) via RT−qPCR. (**D**) Correlation analysis between circCDKN2B−AS_006 and miR−1258 expression in the synovia from RA patients. (**E**) RT−qPCR analysis of the relative expression levels of miR−1258 in RASFs, MH7A cells, and NSFs. (**F**–**H**) RIP with an anti−AGO2 antibody in RASFs and MH7A cells was used to detect the circCDKN2B−AS_006 and miR−1258 mRNA levels. (**I**) RNA pull−down was executed in RASFs and MH7A cells, followed by RT−qPCR to detect the enrichment of circCDKN2B−AS_006. (**J**) A schematic of the wild−type (WT) and mutant (MUT) circCDKN2B−AS_006 luciferase reporter vectors. (**K**) The luciferase activities of the circCDKN2B−AS_006 luciferase reporter vector (WT or MUT) in 293T cells transfected with miR−1258 mimics or NC mimics. (**L**) The expression of miR−1258 in RASFs and MH7A cells after knocking down and overexpressing circCDKN2B−AS_006. (**M**) FISH results, showing the colocalization of circCDKN2B−AS_006 and miR−1258 in RASFs and MH7A cells. ** *p* < 0.01.

CircRNAs have been reported to function as miRNA sponges to competitively bind miRNAs and regulate downstream gene expression [15]. According to the results of whole−transcriptome sequencing, there were 138 differentially expressed miRNAs with a cut-off criteria of fold change > 2.0 and *p* < 0.05, including 72 upregulated miRNAs and 66 downregulated miRNAs (Figure 3A). To determine which miRNAs interact with circCDKN2B−AS_006, a candidate miRNA (miR-1258) was predicted through overlapping of the results of searching for miRNA response elements (MREs) in the circCDKN2B−AS_006 sequence obtained from the public database (circular RNA interactome) and the results of whole−transcriptome sequencing (downregulated miRNAs) (Figure 3B). Next, the expression of miR−1258 was measured in the synovium samples from 40 RA patients and 16 non−RA patients. The results showed that the expression of miR−1258 was significantly decreased in the synovia from RA patients compared with patients with joint trauma (Figure 3C). Pearson analysis showed a negative relationship between the expression of circCDKN2B−AS_006 and miR−1258 in the synovia from RA patients (Figure 3D). Consistent with these findings, miR−1258 was significantly downregulated in RASFs and MH7A cells compared with NSFs (Figure 3E). In general, miRNAs function through the RNA−induced silencing complex (RISC), binding to Ago2. Accordingly, an anti−Ago2 RIP assay was performed, and the results showed that both circCDKN2B−AS_006 and miR−1258 were pulled down by the anti−Ago2 antibody but not by IgG (Figure 3F–H). To further verify the binding of circCDKN2B−AS_006 and miR-1258, an miR pull-down assay with a specific biotin-labeled miR−1258 probe was performed. RT−qPCR analysis revealed the specific enrichment of circCDKN2B−AS_006 in the miR-1258 probe group compared with control probe (Figure 3I). A dual−luciferase reporter assay was performed to identify the specific binding region between circCDKN2B−AS_006 and miR−1258. Based on the complementary base−pairing prediction with the “seed” region of miR−1258, we mutated the predicted binding site of circCDKN2B−AS_006 and inserted the mutant sequence downstream of the luciferase reporter gene (Figure 3J). A dual−luciferase reporter assay revealed that the luciferase activity was significantly decreased in 239T cells after co−transfecting with miR−1258 mimics and a circCDKN2B−AS_006−WT reporter gene, whereas no significant difference was observed after the co−transfection of miR−1258 mimics or NC mimics with mutant reporters (Figure 3K). CircCDKN2B−AS_006 in RASFs and MH7A cells negatively regulated the expression of miR-1258 after circCDKN2B−AS_006 knockdown or overexpression (Figure 3L). FISH assays showed that circCDKN2B−AS_006 colocalized with miR−1258 in the cytoplasm of RASFs and MH7A cells (Figure 3M). These data demonstrate that circCDKN2B−AS_006 acts as a sponge of miR−1258 and suppresses its expression.

### 2.4. MiR−1258 Reverses circCDKN2B−AS_006−Promoted Cell Proliferation, Migration, and Invasion of RASFs and MH7A Cells

It has been reported that miR−1258 has an anti-cancer role in various tumors [16,17]. Thus, we assessed the role of miR−1258 in RASFs and MH7A cells. CCK−8 and EdU assays showed that miR−1258 mimics decreased the proliferation of RASFs (Supplementary Figure S2A,B,E) and MH7A cells (Supplementary Figure S3A,B,E), whereas miR−1258 inhibitors increased cell growth. In the transwell and wound healing assays, we found that miR−1258 mimics decreased the migration and invasion of RASFs (Supplementary Figure S2C,D,F) and MH7A cells (Supplementary Figure S3C,D,F), whereas miR−1258 inhibitors had the opposite effect. These results indicate that miR-1258 inhibits the cell proliferation, migration, and invasion of RASFs and MH7A cells.

**Figure 4:**
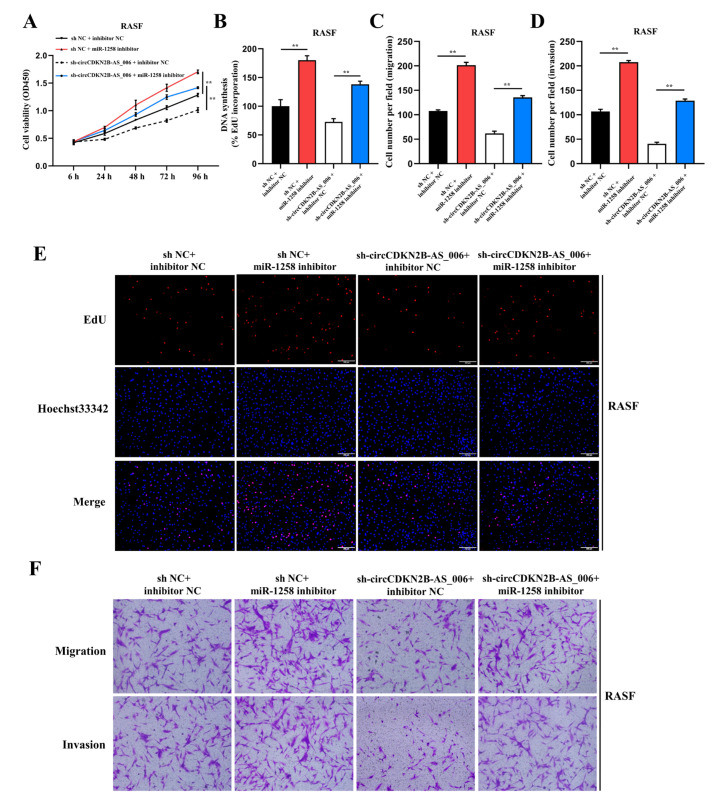
MiR−1258 reverses circCDKN2B−AS_006-promoted cell proliferation, migration, and invasion of RASFs. (**A**,**B**,**E**) The cell proliferation levels were determined after transfection with sh−circCDKN2B−AS_006 and miR−1258 inhibitors via the CCK−8 (**A**) and EdU assays (**B**,**E**). (**C**,**D**,**F**) After transfection with sh−circCDKN2B−AS_006 and miR−1258 inhibitors, the cell migration and invasion of RASFs were determined by means of transwell assay. ** *p* < 0.01.

To elucidate whether circCDKN2B−AS_006 functions by sponging miR−1258, rescue experiments were performed with the co−transfection of miR−1258 inhibitors with LV−sh−circCDKN2B−AS_006. The results indicated that miR−1258 inhibitors significantly reversed the decreased proliferation, migration, and invasion induced by circCDKN2B−AS_006 knockdown in the CCK−8 (Figure 4A), EdU (Figure 4B,E), and transwell (Figure 4C,D,F) assays in RASFs. Similar results were observed after transfection with miR−1258 inhibitors and LV−sh−circCDKN2B−AS_006 in MH7A cells (Supplementary Figure S4). Collectively, these data demonstrate that miR−1258 serves a crucial function downstream of circCDKN2B−AS_006.

### 2.5. RUNX1 Is a Downstream Target of miR−1258

**Figure 5:**
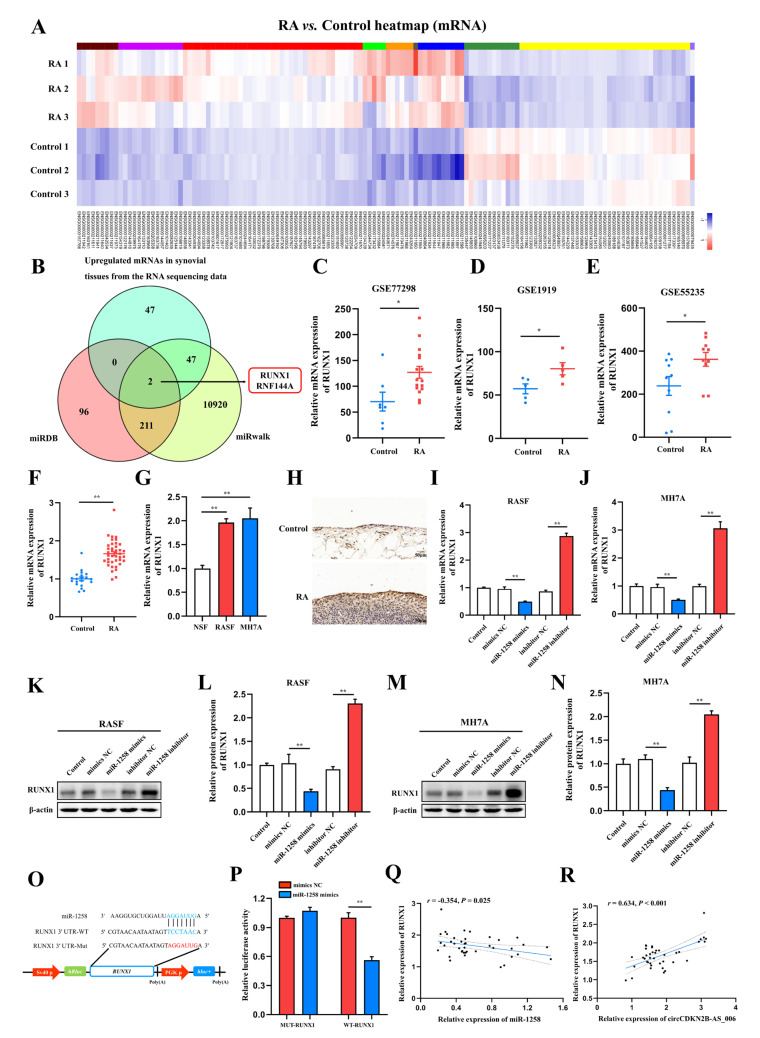
RUNX1 is a direct target of miR−1258. (**A**) Expression heatmap of differentially expressed mRNAs in two groups. (**B**) Venn diagram of the numbers of overlapping targeted mRNAs of miR−1258 predicted by means of two algorithms (miRDB and miRWalk) and the results of whole−transcriptome sequencing (upregulated mRNAs). (**C**–**E**) RUNX1 expression was significantly downregulated in the synovia from RA patients according to the NCBI GEO database (GSE77298, GSE1919, and GSE55235). (**F**) The relative expression of RUNX1 was detected in the synovia from RA patients (*n* = 40) and non-RA patients (*n* = 16) by means of RT−qPCR. (**G**) RT−qPCR analysis of the relative expression levels of RUNX1 in RASFs, MH7A cells, and NSFs. (**H**) Immunohistochemistry staining showing the protein expression of RUNX1 in the synovia from RA patients and non−RA patients. (**I**,**J**) The mRNA expression of RUNX1 in RASFs and MH7A cells transfected with miR−1258 mimics or inhibitors. (**K**–**N**) The protein expression of RUNX1 in RASFs and MH7A cells transfected with miR−1258 mimics or inhibitors. (**O**) Schematic of the RUNX1 3′ UTR wild−type (WT) and mutant (MUT) luciferase reporter vectors. (**P**) Dual−luciferase reporter assays were carried out to detect the relative luciferase activity of luciferase reporter plasmid containing the RUNX1 3′ UTR wild−type (WT) or mutant (MUT), which were cotransfected with the miR−1258 mimics or NC mimics. (**Q**) Correlation analysis between miR−1258 and RUNX1 expression in the synovia from RA patients. (**R**) Correlation analysis between circCDKN2B−AS_006 and RUNX1 expression in the synovia from RA patients. ** *p* < 0.01, * *p* < 0.05.

MiRNAs interact with the 3′ untranslated region (3′ UTR) of target genes to regulate their expression [18]. According to the results of whole−transcriptome sequencing, a total of 185 differentially expressed miRNAs with a cut−off criteria of fold change > 2.0 and *p* < 0.05 were found, including 100 upregulated miRNAs and 85 downregulated miRNAs (Figure 5A). To further investigate the target genes of miR−1258, two candidate mRNAs, namely, runt−related transcription factor 1 (RUNX1) and ring finger protein 144A (RNF144A), were predicted through overlapping the results of searching for the potential target genes of miR−1258 obtained from two public databases (miRDB and miRwalk) and the results of whole−transcriptome sequencing (upregulated mRNAs) (Figure 5B). RUNX1 has been confirmed to act as an oncogene in a variety of cancers [19]. The expression of RUNX1 in the synovia from patients with RA was further bioinformatically analyzed based on microarray expression data from the NCBI GEO database (GSE77298, GSE1919, and GSE55235). The results showed that the expression of RUNX1 in the synovia of patients with RA in GSE77298 (Figure 5C), GSE1919 (Figure 5D), and GSE55235 (Figure 5E) was higher compared with normal controls. Consistently, the expression of RUNX1 was significantly increased in the synovia from 40 RA patients compared with patients with joint trauma (Figure 5F). RUNX1 was significantly upregulated in RASFs and MH7A cells compared with NSFs (Figure 5G). The results of immunohistochemistry staining showed that the protein expression of RUNX1 was upregulated in the synovia from RA patients in comparison to those from patients with joint trauma (Figure 5H).

Since miRNAs typically downregulate the expression of target genes by binding to their 3′ UTRs, the expression of RUNX1 in RASFs and MH7A cells using miR−1258 mimics or inhibitors with RT−qPCR and Western blot assays was measured. The RT−qPCR and Western blot results identified that miR−1258 mimics downregulated the RUNX1 expression at both the mRNA and protein levels in RASFs (Figure 5I,K,L) and MH7A cells (Figure 5J,M,N).

Next, the wild−type and mutant dual−luciferase reporter plasmids harboring the wild−type or mutant RUNX1 3′ UTR were constructed (Figure 5O), and the results showed that the transfection of 293T cells with miR−1258 mimics notably downregulated the luciferase activity in the plasmid with the WT but not the MUT 3′ UTR of RUNX1 (Figure 5P). Pearson analysis showed that the expression of RUNX1 was negatively correlated with the expression of miR−1258 (Figure 5O), whereas it was positively correlated with the expression of RUNX1 (Figure 5R) in the synovia from RA patients.

### 2.6. CircCDKN2B−AS_006 Promotes RASF and MH7A Proliferation, Migration, and Invasion via the miR−1258/RUNX1 Axis

**Figure 6:**
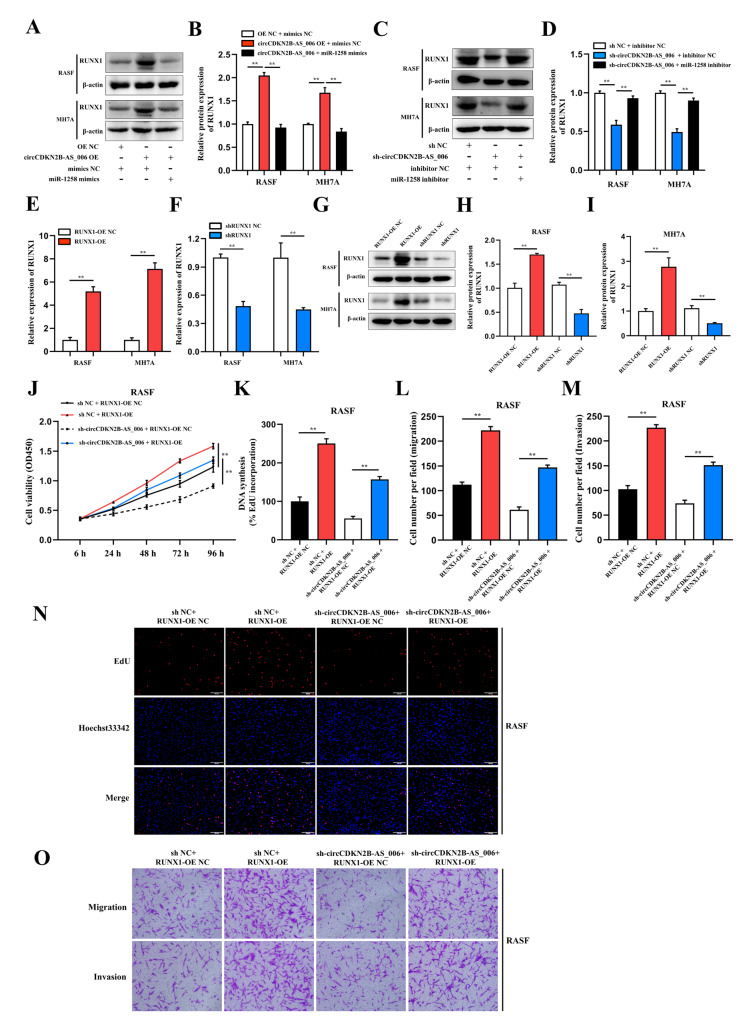
CircCDKN2B−AS_006 promotes RASF proliferation, migration, and invasion via the miR−1258/RUNX1 axis. (**A**,**B**) RUNX1 expression was analyzed in RASFs and MH7A cells after transfection with circCDKN2B−AS_006 overexpression and miR−1258 mimics. (**C**,**D**) RUNX1 expression was analyzed in RASFs and MH7A cells after transfection with circCDKN2B−AS_006 knockdown and miR−1258 inhibitors. (**E**,**F**) The efficiency of RUNX1 overexpression (**E**) and silencing (**F**) in RASFs and MH7A cells was verified by means of RT−qPCR. (**G**–**I**) The efficiency of RUNX1 overexpression (**G**,**H**) and silencing (**G**,**I**) in RASFs and MH7A cells was verified by means of Western blot analysis. (**J**,**K**,**N**) The cell proliferation levels were determined after transfection with sh−circCDKN2B−AS_006 and RUNX1 overexpression by means of the CCK−8 (**J**) and EdU assays (**K**,**N**). (**L**,**M**,**O**) After transfection with sh−circCDKN2B−AS_006 and RUNX1 overexpression, the cell migration and invasion were determined by transwell assay. ** *p* < 0.01.

As RUNX1 is a structural target that functions downstream of miR−1258 and circCDKN2B−AS_006, we further evaluated whether circCDKN2B−AS_006 and RUNX1 are functionally associated. Overexpression of circCDKN2B−AS_006 significantly increased the protein levels of RUNX1 and the effect could be abolished by miR−1258 mimics (Figure 6A,B). Conversely, knockdown of circCDKN2B−AS_006 significantly decreased the protein levels of RUNX1 and the effect could be abolished by miR−1258 inhibitors (Figure 6C,D). To regulate the RUNX1 mRNA and protein levels, RUNX1−overexpression lentivirus and shRNA lentivirus were constructed and transfected into RASFs and MH7A cells (Figure 6E–I). Subsequently, the CCK−8 and EdU assays showed that RUNX1 overexpression significantly reversed the decreased proliferation induced by circCDKN2B−AS_006 knockdown in RASFs (Figure 6J,K,N) and MH7A cells (Supplementary Figure S5A,B,E). The transwell assay showed that RUNX1 overexpression significantly reversed the decreased migration and invasion induced by circCDKN2B−AS_006 knockdown in RASFs (Figure 6L,M,Q) and MH7A cells (Supplementary Figure S5C,D,F). Collectively, our results suggested that circCDKN2B−AS_006 promoted the proliferation, migration, and invasion of RASFs and MH7A cells via the miR−1258/RUNX1 axis.

### 2.7. RUNX1 Promotes EMT and Enhances Wnt/β−Catenin Pathway Activation

The role of RUNX1 in tumor metastasis has been examined in several studies, and its possible mechanism involves activating the Wnt/β−catenin signaling pathway and EMT [20,21]. As RUNX1 was able to stimulate the proliferation, migration, and invasion of RASFs and MH7A cells, we further explored whether the role of RUNX1 in RASF and MH7A was related to the Wnt/β−catenin signaling pathway and EMT. GSEA analysis using the GSE77298, GSE55235, and GSE55584 databases showed that *RUNX1* expression was associated with ECM receptor interaction, adherens junctions, the TGF−β signaling pathway, focal adhesion, and the Wnt signaling pathway (Supplementary Figure S6A–C). We retrieved genes with *RUNX1* transcription factor binding sites from the TRANSFAC Predicted Transcription Factor Targets database and used the results to conduct a KEGG pathway analysis using DAVID Bioinformatics Resources 6.8. The KEGG pathway analysis results showed gene enrichment in focal adhesion, pathways in cancer, ECM receptor interaction, the TGF−β signaling pathway, and the Wnt signaling pathway (Supplementary Figure S6D). Next, an upset Venn diagram was used to identify the overlapping genes among the KEGG pathways related to the EMT process, the Wnt/β−catenin pathway, and cancer; *CTNNB1*, also known as β−catenin, was the intersection gene of each pathway (Supplementary Figure S6E). Subsequently, a positive correlation was observed between the expression of *RUNX1* and *CTNNB1* in the gene expression microarray dataset (GSE55235) in the synovia from RA patients (Supplementary Figure S6F).

**Figure 7:**
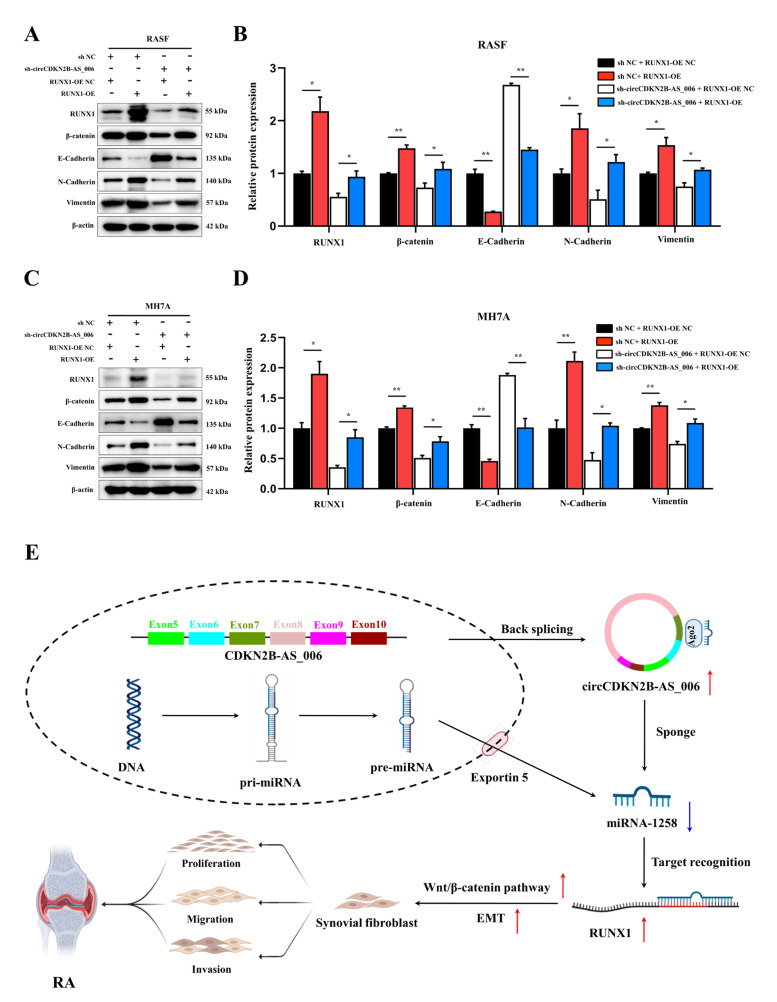
RUNX1 promotes EMT and enhances Wnt/β−catenin pathway activation. (**A**,**B**) RUNX1, β−catenin, and EMT−related protein expression was analyzed in RASFs after transfection with sh−circCDKN2B−AS_006 and RUNX1−OE. (**C**,**D**) RUNX1, β−catenin, and EMT−related protein expression was analyzed in MH7A cells after transfection with sh−circCDKN2B−AS_006 and RUNX1−OE. (**E**) Schematic diagram illustrating the mechanism by which circCDKN2B−AS_006 promotes RASF proliferation, migration, and invasion through miR−1258/Wnt/β−catenin pathway and EMT axis. ** *p* < 0.01, * *p* < 0.05.

As shown in Figure 7A–D, the Western blot analysis results demonstrated that RUNX1 upregulation enhanced the protein levels of β−catenin, N−cadherin, and vimentin, and decreased the protein levels of E−cadherin. Moreover, RASFs and MH7A cells with the depletion of circCDKN2B−AS_006 exhibited higher levels of N−cadherin and lower expression of β−catenin, E−cadherin, and vimentin than the sh NC + RUNX1−OE NC group, which could be rescued by RUNX1 overexpression. These results indicated that circCDKN2B−AS_006 regulated the expression of RUNX1, leading to the activation of the Wnt/β−catenin signaling pathway and EMT.

### 2.8. Local Knockdown of circCDKN2B−AS_006 Attenuated the Severity of CIA In Vivo

**Figure 8:**
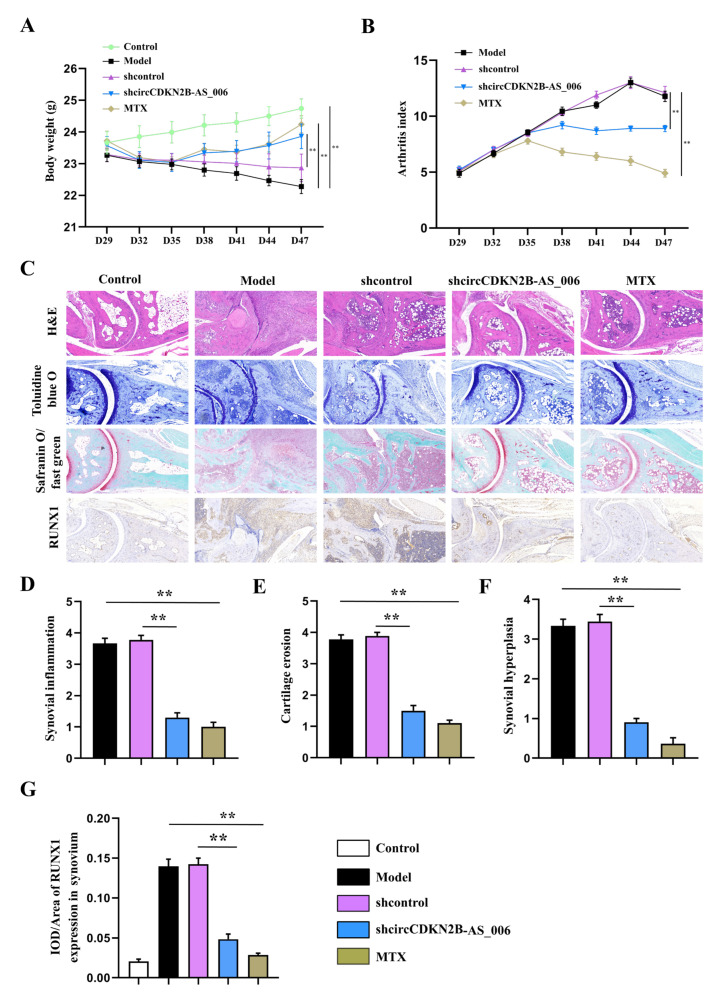
Local knockdown of circCDKN2B−AS_006 attenuated the severity of CIA in mice. (**A**) Effects of sh−circCDKN2B−AS_006 on the body weight of CIA mice. (**B**) Effects of sh−circCDKN2B−AS_006 on the polyarthritis index of CIA mice. (**C**) Representative micrographs of HE-, toluidine blue−, and safranin O/fast green−stained histological sections and immunostaining of RUNX1 and β−catenin of the joints. (**D**) Quantification of the histomorphometric analysis of synovial inflammation. (**E**) Quantification of the histomorphometric analysis of cartilage damage. (**F**) Quantification of the histomorphometric analysis of bone erosion. (**G**) Quantitative analysis of the immunohistochemical detection of RUNX1 expression in synovia. ** *p* < 0.01 compared to the shcontrol group (*n* = 9–10 per group).

To determine whether circCDKN2B−AS_006 plays a crucial pathogenic role in the progression of RA, the lentivirus of circCDKN2B−AS_006 shRNA was locally injected into inflamed joints to knock down the expression of circCDKN2B−AS_006 in CIA mice. As shown in Figure 8A,B, administration of lentivirus expressing shRNA for circCDKN2B−AS_006 (sh−circCDKN2B−AS_006) in CIA mice significantly increased the body weight and decreased the arthritic index compared with CIA mice receiving control shRNA injections (shcontrol). Similarly, administration of positive drug (MTX) in CIA mice also increased the body weight and decreased the arthritic index compared with model mice (Figure 8A,B). The severity of arthritis in the CIA mice was further evaluated via histological analysis (H&E, toluidine blue O, and safranin O/fast green staining) of ankle joint sections (Figure 8C). Synovial hyperplasia and massive joint destruction with the erosion of cartilage were observed in the ankle joints of the CIA mice. There was significant decreased synovial inflammation, as well as cartilage and bone destruction, in mice after the administration of sh−circCDKN2B−AS_006 or MTX compared with shcontrol group or model group, respectively (Figure 8D–F). Moreover, immunohistochemical staining of joint tissue sections (Figure 8C) and average integrated optical density (IOD) values (Figure 8G,H) showed the increased staining intensity of RUNX1 and β−catenin in CIA mice compared with that observed in normal control mice, which could be reduced by sh−circCDKN2B−AS_006 injection treatment or MTX treatment. Taken together, these findings indicate that knockdown of circCDKN2B−AS_006 suppresses arthritic progression, with significant amelioration of joint damage.

### 2.9. CircCDKN2B−AS_006/miR−1258/RUNX1 Is Correlated with Clinical Features of RA

**Figure 9:**
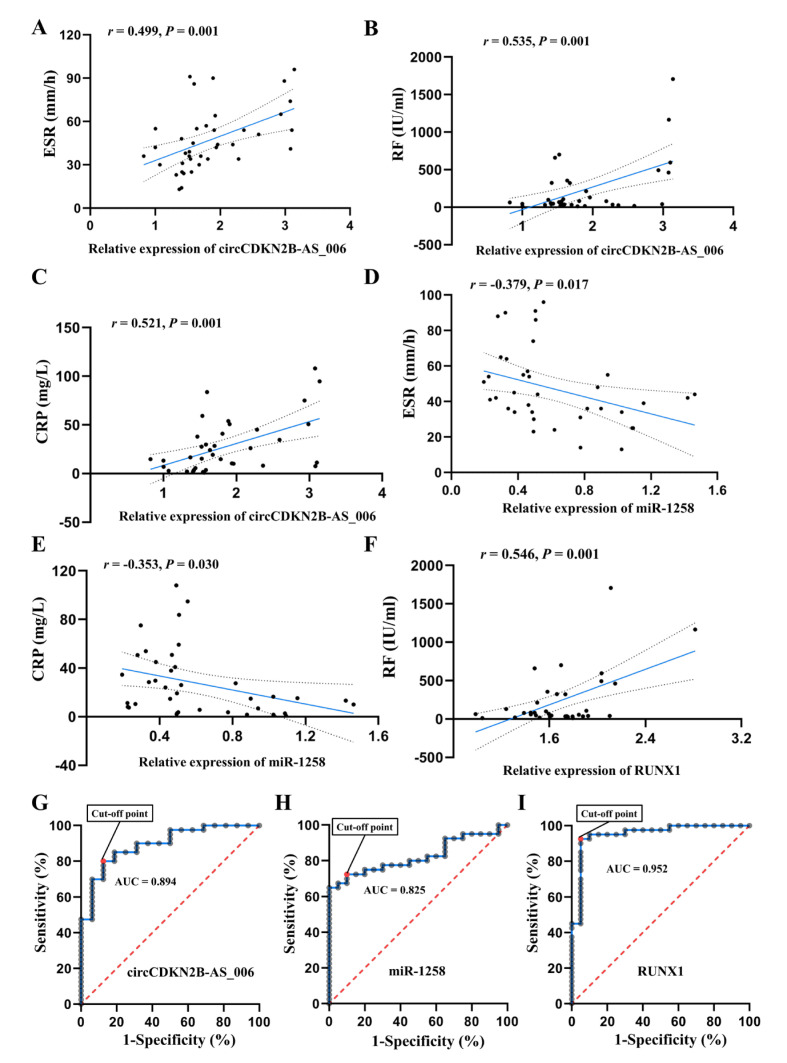
The circCDKN2B−AS_006/miR−1258/RUNX1 axis is correlated with the clinical indicators of RA patients. (**A**) Correlation between circCDKN2B−AS_006 expression in synovia and ESR in patients with RA. (**B**) Correlation between circCDKN2B−AS_006 expression in synovia and serum RF levels in patients with RA. (**C**) Correlation between circCDKN2B−AS_006 expression in synovia and serum CRP levels in patients with RA. (**D**) Correlation between miR−1258 expression in synovia and ESR in patients with RA. (**E**) Correlation between miR−1258 expression in synovia and serum CRP levels in patients with RA. (**F**) Correlation between RUNX1 expression in synovia and serum RF levels in patients with RA. (**G**) ROC curve of circCDKN2B−AS_006 expression in synovia in discriminating patients with RA from patients with joint trauma. (**H**) ROC curve of miR−1258 expression in synovia in discriminating patients with RA from patients with joint trauma. (**I**) ROC curve of RUNX1 expression in synovia in discriminating patients with RA from patients with joint trauma.

The relationship between circCDKN2B−AS_006, miR−1258, and RUNX1 expression and the clinical characteristics of the RA patients are listed Figure 9A–F. For the statistical analysis, patients with RA were divided into high and low expression groups with regard to circCDKN2B−AS_006/miR−1258/RUNX1, according to a 50th percentile cutoff. RA patients with high circCDKN2B−AS_006 expression had increased serum CRP levels and ESR compared to RA patients with low circCDKN2B−AS_006 expression, whereas RA patients with high miR−1258 expression exhibited decreased serum CRP levels and ESR compared to RA patients with low miR−1258 expression. Pearson correlation analysis showed that circCDKN2B−AS_006 expression was positively correlated with erythrocyte sedimentation rates (ESR) (Figure 9A), serum rheumatoid factor (RF) (Figure 9B), and C−reactive protein (CRP) (Figure 9C) levels, whereas miR−1258 expression was negatively correlated with ESR (Figure 9D) and serum CRP (Figure 9E) levels. Moreover, a positive relationship was found between RUNX1 expression and serum RF levels (Figure 9F). Furthermore, ROC analysis indicated the potential diagnostic value of circCDKN2B−AS_006 (Figure 9G), miR−1258 (Figure 9H), and RUNX1 (Figure 9I) expression in synovium samples in discriminating patients with RA from patients with joint trauma, with AUC values of 0.894, 0.825, and 0.952, respectively. These data suggest that circCDKN2B−AS_006, miR−1258, and RUNX1 dysregulation are related to the clinical indicators and disease severity of patients with RA.

## 3. Discussion

Advances in high−throughput sequencing and associated bioinformatics tools and databases have extended the recognition and understanding of circRNAs. Recent studies have indicated that circRNAs may play crucial roles in the progression of RA [22]. It has been reported that the knockdown of circRNA_09505 in macrophages significantly alleviates arthritis and inflammation in vivo in CIA mice [23]. Another study confirmed that circ_0088036 promoted RA progression by acting as a miR−140−3p sponge to upregulate silent information regulator 1 expression [24]. However, fewer studies have been conducted on the roles and underlying mechanisms of circRNAs in the proliferation, migration, and invasion of activated RASFs. Through a series of experiments, our investigation was the first to reveal the existence of the circCDKN2B−AS_006/miR−1258/RUNX1 axis in the progression of RA (Figure 7E).

In the present study, we profiled circRNA expression in synovia from three pairs of RA patients and non−RA patients by means of RNA sequencing, resulting in the identification of 539 differentially expressed circRNAs. For the first time, we identified circCDKN2B−AS_006 as a significantly upregulated circRNA in synovia from RA patients. Loss− and gain−of−function experimental results suggested that circCDKN2B−AS_006 promotes the proliferation, migration, and invasion of RASFs in vitro. Moreover, circCDKN2B−AS_006 knockdown alleviated the severity of arthritis in the CIA mice model. In addition, circCDKN2B−AS_006 expression was positively correlated with ESR, serum RF, and CRP levels in patients with RA. These results indicate the critical role of circCDKN2B−AS_006 in RASF tumor-like growth and metastasis and the prognosis of RA patients.

CircRNAs play vital roles in many physiological and pathological processes, including acting as microRNA (miRNA) sponges, interacting with RNA binding proteins (RBPs), and regulating transcription or translation [25]. Emerging evidence has revealed that circRNAs could function as miRNAs [26]. It has been reported that circLMTK2 inhibits the proliferation and metastasis of gastric cancer by acting as a competitive endogenous RNA through sponging miR-150-5p [27]. Similarly, circBCRC−3 suppresses bladder cancer proliferation by acting as an endogenous miR−182−5p sponge, resulting in the upregulation of p27 [28]. In the present study, through cross-analysis of a miRNA target prediction database (circular RNA interactome) and the results of whole−transcriptome sequencing (down−regulated miRNAs), we initially identified miR−1258 as a candidate target of circCDKN2B−AS_006. Pull−down, anti−Ago2 RIP, dual−luciferase, and FISH assays were conducted to validate the binding between circCDKN2B−AS_006 and miR−1258. The results indicate that, as an RISC complex, circCDKN2B−AS_006 could directly bind to the seed region of miR−1258 in the cytoplasm of RASFs and MH7A cells. Moreover, to validate whether circCDKN2B−AS_006 could exert biological functions via miR−1258, we designed rescue experiments using miR−1258 inhibitors, and the results showed that the circCDKN2B−AS_006 knockdown-induced suppression of cell proliferation, migration, and invasion was reversed through treatment with a miR−1258 inhibitor. Our results provide evidence to support the conjecture that circCDKN2B−AS_006 functions as a miRNA sponge that is important for the progression and metastasis of RASFs.

Previous studies have confirmed miR−1258 as a tumor suppressor in multiple cancers, including breast cancer [29], hepatocellular carcinoma [30], non−small−cell lung cancer [16], and gastric cancer [31]. Specifically, it has been demonstrated that miR−1258 can target heparanase and subsequently inhibit the cell−aggressive phenotype in breast cancer [17]. Moreover, miR−1258 may play tumor−suppressive roles by targeting cyclin−dependent kinase regulatory subunit 1B expression in colorectal cancer [32]. In the present study, we first found that miR−1258 was downregulated in the synovia from RA patients. Gain− and loss−of−function experiments showed that miR−1258 inhibited the proliferation, migration, and invasion of RASFs and MH7A cells, suggesting that miR−1258 functioned as a suppressor in RASF tumor−like growth and metastasis and the prognosis of RA patients.

MiRNAs are small RNAs of 21−25 nucleotides that bind to partially complementary sequences in the 3′ UTR of mRNA and negatively affect post−transcriptional regulation [33]. To further elucidate the underlying mechanism of miR−1258, we conducted bioinformatic, RT−qPCR, Western blot, and dual−luciferase analyses, with the results identifying RUNX1 as the most likely target of miR−1258. RUNX1, also called acute myeloid leukaemia 1, is a member of the RUNX family of transcription factors (RUNX1, RUNX2, and RUNX3), and this family is composed of evolutionarily conserved transcription factors that function as critical lineage determinants in various tissues [34]. It has been reported that the suppression of RUNX1 inhibited cell proliferation, migration, and invasion in head and neck squamous cell carcinoma (HNSCC) [35]. On the contrary, RUNX1 activation promoted colorectal cancer cell proliferation [36]. In the present study, after constructing RUNX1 shRNA and overexpression lentiviral constructs, we observed that RUNX1 could remarkably promote the proliferation, migration, and invasion of RASFs and MH7A cells. Moreover, the results of rescue experiments showed that RUNX1 overexpression could mediate the inhibitory effect of circCDKN2B−AS_006 knockdown on cell proliferation, migration, and invasion. Taken together, our results revealed that circCDKN2B−AS_006 promoted RASF and MH7A proliferation, migration, and invasion via the miR−1258/RUNX1 axis.

Accumulating evidence has demonstrated that RUNX1 is closely associated with the EMT process and the Wnt/β−catenin signaling pathway. A recent study showed that RUNX1 promotes colorectal cancer metastasis by activating the Wnt/β−catenin signaling pathway and EMT [23]. Another study showed that RUNX1 promoted renal tubular EMT and kidney fibrosis by regulating TGF−β [37]. In the present study, through bioinformatic analyses, we initially found that RUNX1 was related to the Wnt/β−catenin signaling pathway and EMT in the synovia from RA patients. Moreover, the Western blot results showed that the overexpression of RUNX1 significantly increased the levels of β−catenin and the EMT−related proteins N−cadherin and vimentin, and decreased the levels of E−cadherin. Furthermore, knockdown of circCDKN2B−AS_006 decreased the levels of β−catenin, N−cadherin, and vimentin, and increased the levels of E−cadherin, which could be reversed via RUNX1 overexpression. Collectively, these data suggest that circCDKN2B−AS_006 promotes RASF and MH7A proliferation, migration, and invasion by activating the Wnt/β−catenin signaling pathway and EMT via the miR−1258/RUNX1 axis.

Further results revealed that the knockdown of circCDKN2B−AS_006 inhibited synovial proliferation, inflammatory cell infiltration, and articular cartilage and bone erosion in CIA mice. Immunohistochemistry staining results confirmed that downregulation of circCDKN2B−AS_006 inhibited the protein expressions of RUNX1 and β−catenin. These results indicated that knockdown of circCDKN2B−AS_006 suppressed arthritic progression with significant amelioration of joint damage via the RUNX1/β−catenin axis.

We further explored the relationship between circCDKN2B−AS_006, miR−1258, and RUNX1 expression and the clinical characteristics of RA patients. Blood biomarkers including RF, anti−cyclic citrullinated peptide antibodies (ACPA), antistreptolysin O (ASO), ESR, and CRP have shown good performance in discriminating among a portion of typical RA patients and predicting rapid progression [38,39,40]. The 28−joint−count disease activity score (DAS−28) incorporates one of two inflammatory markers, ESR or CRP [41], and is an assessment used to measure the level of disease activity in patients with RA [42]. RF and ACPA are immunological hallmarks of RA, and the presence of these antibodies in RA is associated with higher disease activity and an increased risk of joint destruction [43]. Therefore, clinical features including X−ray classification, time of pain, ACPA, ASO, RF, ESR, and CRP were used to evaluate the disease severity of RA in the present study. The expression of circCDKN2B−AS_006/miR−1258/RUNX1 was associated with ESR, CRP, and RF levels in serum from RA patients, suggesting a significant relationship between circCDKN2B−AS_006/miR−1258/RUNX1 expression and RA disease activity. In addition, the results of ROC curve analysis showed that the expression of circCDKN2B−AS_006/miR−1258/RUNX1 in synovium samples may represent a useful tool for discriminating patients with RA from patients with joint trauma.

Several limitations should be highlighted. Firstly, the extracellular environment of RASF in the joint cavity of RA patients is inflammatory joint fluid, while the extracellular environment of RASF in the present study is normal cell culture medium. It may be more appropriate to use freshly isolated synovial fluid from patients with RA as a culture medium for RASF. Secondly, only one of the differentially expressed circRNAs was investigated in the present study. Thirdly, patients with joint trauma were selected as control group. Given that these patients with joint trauma may have acute inflammation in the joint, normal subjects (post-mortem joint samples) may be more suitable as control group.

In summary, circCDKN2B−AS_006 was upregulated in the synovia from RA patients and was positively associated with the clinical features of RA. Functionally and mechanistically, the circCDKN2B−AS_006/miR−1258/RUNX1 axis promoted the proliferation, migration, and invasion of RASFs through the activation of the Wnt/β−catenin pathway and EMT, suggesting a potentially promising therapeutic target for RA.

## 4. Materials and Methods

### 4.1. Patients and Tissues

Patients who fulfilled the American College of Rheumatology criteria for RA were invited to enroll in the present study. The control group consisted of age− and gender−matched patients with joint trauma. Synovium samples from patients with RA (RA group; *n* = 40) and patients with joint trauma (control group; *n* = 16) were collected during joint surgery at the First Affiliated Hospital of Anhui Medical University. Patients who had a history of autoimmune or infectious disease were excluded from the control group. All participants read and signed written informed consent forms before voluntary participation. This study complied with the guidelines of the Declaration of Helsinki and was authorized by the Human Research Ethics Committee of Anhui Medical University (20210331).

### 4.2. RNA Extraction, Library Construction, and Sequencing

The synovium samples were submitted to the Majorbio Bio−pharm Technology Co., Ltd. (Shanghai, China) for total RNA extraction, mRNA isolation and purification, sequencing library preparation, and sequencing. Briefly, the total RNA was isolated from the synovia of RA patients or control subjects using TRIzol reagent (Invitrogen, Waltham, MA, USA) according to the manufacturer’s instructions. A NanoDrop ND−1000 spectrophotometer (NanoDrop Technologies, Wilmington, DE, USA) was used to determine RNA purity and concentrations. A 2100 Bioanalyzer (Agilent Technologies, Santa Clara, CA, USA) and an ND−2000 (NanoDrop Technologies, Wilmington, DE, USA) were used to determine the RNA quality. The ribosomal RNA was digested from the total RNA using the Ribo−Zero Gold kit (Illumina, San Diego, CA, USA). The libraries were then constructed and sequenced on an Illumina HiSeq xten system. Differential mRNA, miRNA, and circRNA expression analysis of the two groups was conducted via DEGseq. Fold change (FC) was regarded as an indicator of differential expression between the two groups. T−tests were utilized to evaluate the statistical significance of differences. *p*-values < 0.05 were considered to demonstrate differential expression.

### 4.3. Isolation and Culture of SF

SFs were isolated from the synovia of RA patients (RASF) or control subjects (normal synovial fibroblasts (NSFs)) as described previously [44]. Briefly, fresh knee synovial tissues were divided into very small pieces (1 mm^3^) using scissors or were minced under sterile conditions. The tissues were gently pipetted using a Pasteur pipette and attached to the wall of the cell culture flask and cultured in DMEM/high−glucose medium (Hyclone, Logan, UT, USA) containing 20% (*v*/*v*) fetal bovine serum (FBS) (Gibco, Grand Island, NY, USA), 100 mg/mL of streptomycin, and 100 U/mL of penicillin (both from Beyotime, Shanghai, China). The culture flask was placed upright in a 37 °C incubator in 5% CO_2_ atmosphere for 6 h to allow tissue adhesion. After the formation of fibroblast−like cell colonies, adherent cells were split. All experiments were performed using synoviocyte cultures from the 4th to the 7th passages.

### 4.4. Cell Culture

RASFs and NSFs were cultured in DMEM/high−glucose medium (Hyclone, Logan, UT, USA) containing 20% (*v*/*v*) fetal bovine serum (FBS) (Gibco, Grand Island, NY, USA), 100 mg/mL of streptomycin, and 100 U/mL of penicillin (both from Beyotime, Shanghai, China) at 37 °C in a humidified atmosphere containing 95% air and 5% CO_2_.

MH7A cells, a human rheumatoid arthritis synovial cell line, were obtained from Jennio Biotech Co., Ltd. (Guangzhou, China). The cells were incubated in DMEM/high-glucose medium (Hyclone, Logan, UT, USA) containing 10% (*v*/*v*) fetal bovine serum (FBS) (Gibco, Grand Island, NY, USA), 100 mg/mL of streptomycin, and 100 U/mL of penicillin (both from Beyotime, Shanghai, China) at 37 °C in a humidified atmosphere containing 95% air and 5% CO_2_.

### 4.5. Oligonucleotides (Oligos), Plasmids, and Cell Transfection

The circCDKN2B−AS_006 plasmid, circCDKN2B−AS_006 short hairpin RNA (shRNA) plasmid, miR-1258 mimics, miR−1258 inhibitor, RUNX1 plasmid, RUNX1 shRNA plasmid, and the corresponding controls were provided by GenePharma (Shanghai, China). The circCDKN2B−AS_006 overexpression plasmid (circCDKN2B−AS_006 OE) or RUNX1 overexpression plasmid (RUNX1−OE) was generated by inserting the full−length circCDKN2B−AS_006 or RUNX1 sequence into the LV17 (EF−1a/Luciferase17&Puro) lentiviral vector or LV18C (LV18−CMV−T2A−Neo) lentiviral vector, respectively (GenePharma, Shanghai, China). The shRNA plasmid targeting the back−splice junction of circCDKN2B−AS_006 (sh−circCDKN2B−AS_006) or RUNX1 (shRUNX1) was synthesized and cloned into the LV16 (U6/Luciferase17&Puro) lentiviral vector or LV (LV−U6−copGFP−T2A−Neo) lentiviral vector, respectively (GenePharma, Shanghai, China). For the preparation of lentivirus, HEK−293 T cells were co−transfected with a lentiviral vector plus packaging plasmids using Lipofectamine 3000 reagent (Invitrogen, Carlsbad, CA, USA). RASFs or MH7A cells were infected with the packaged lentivirus and then selected with 2 μg/mL puromycin and/or 400 μg/mL geneticin (G418) (Sigma, St. Louis, MO, USA). Cell transfection with the miRNA mimics, miRNA inhibitors, or vectors was performed using Lipofectamine 3000 (Invitrogen, Carlsbad, CA, USA). All the sequences used in the present study are listed in Table S1.

### 4.6. Quantitative Real−Time Polymerase Chain Reaction (RT−qPCR)

This assay was performed according to a previous study [45]. Total RNA was obtained from the synovia and cultured cells using the TRIzol reagent (Takara, Shiga, Japan) according to the manufacturer’s instructions. Then, total RNA quantification was performed using Nanodrop 2000 (Thermo Fisher Scientific, Waltham, MA, USA) and reverse−transcribed into complementary DNA (cDNA) using a First Strand cDNA Synthesis Kit (Thermo Fisher Scientific, Waltham, MA, USA). The synthesized cDNAs were utilized for RT−qPCR, which was performed using a CFX96 real−time RT−PCR detection system (Bio−Rad, Hercules, CA, USA). The RT−qPCR analyses for mRNAs and circRNAs were conducted with a SYBR Premix Ex Taq kit (TaKaRa Biotechnology, Tokyo, Japan). For the detection of miR-1258, the Bulge−Loop™ hsa−miR−1258 RT−qPCR Primer Set (RiboBio, Guangzhou, China) and the U6 snRNA qPCR Primer Set (RiboBio, Guangzhou, China) were used for the amplification of miR−1258 and U6, respectively. The primers for miR−1258 and U6 were designed by RiboBio Inc. (Guangzhou, China), and the sequences are covered by a patent. GAPDH was used as an internal control for circRNAs and mRNAs, and U6 was employed as an endogenous control for the miRNAs. The Ct values of the samples were calculated and the transcript levels were analyzed using the 2^−ΔΔCt^ method. Supplementary Table S2 shows the details of the primers used for RT−qPCR.

### 4.7. Western Blot Analysis

This assay was performed according to a previous study [45]. Cells were rinsed with ice−cold phosphate-buffered saline (PBS) and lysed using radioimmunoprecipitation (RIPA) buffer containing protease inhibitors and phosphatase inhibitors. A BCA Protein Assay Kit (Beyotime Biotechnology, Shanghai, China) was used to measure the total protein concentrations. Equal amounts of protein samples were prepared in loading buffer and boiled at 100 °C for 10 min, and then the boiled samples were electrophoretically separated via sodium dodecyl sulfate polyacrylamide gel electrophoresis (SDS−PAGE) on a 10% gel and transferred onto polyvinylidene difluoride (PVDF) membranes (Millipore Corp, Billerica, MA, USA). After blocking with 5% nonfat dried milk in Tris−buffered saline (TBS) containing 1% Tween−20 for 2 h at room temperature, the membranes were incubated overnight with specific primary antibodies against Argonaute−2 (Ago2; Abcam, #ab156870), RUNX1 (LS Bio, #LS-C353932), β−catenin (Abcam, #ab223075), E−cadherin (Abcam, #ab40772), N−cadherin (Abcam, #ab76011), vimentin (Abcam, #ab92547), and β−actin (Abcam, #ab8227) at 4 °C overnight. After being washed three times with TBST, the membranes were incubated for 1 h at room temperature with horseradish peroxidase (HRP)−conjugated secondary antibodies (1:10,000 dilution). Protein bands were then detected in the enhanced chemiluminescence (ECL) detection system (Thermo Fisher Scientific Inc, Waltham, MA, USA) and quantification was performed using the ImageJ software.

### 4.8. RNA Fluorescence In Situ Hybridization (FISH)

The Cy3−labeled circCDKN2B−AS_006 probes and FAM−labeled miR−1258 probes were designed and synthesized by GenePharma Co. Ltd. (Shanghai, China). RASFs or MH7A cells were seeded on round coverslips, fixed with 4% paraformaldehyde for 10 min, permeabilized in PBS with 0.5% Triton X−100, and dehydrated in ethanol. FISH probes were diluted (1:50), denatured, equilibrated, and added to cells overnight at 37 °C. Then, slides were incubated with 4′,6−diamidino−2−phenylindole (DAPI) for 10 min at room temperature in the dark. After that, slides were sealed with rubber cement and air−dried in the dark for 30 min. Finally, a laser confocal microscope (LSM800, Carl Zeiss, Germany) was used to observe the fluorescence results. The probe sequences are listed in Supplementary Table S1.

### 4.9. Isolation of Nuclear and Cytoplasmic Fractions

Cytosolic and nuclear fractions of cells were prepared using NE-PER Nuclear and Cytoplasmic Extraction Reagents (Thermo Scientific, Rockford, IL, USA). Briefly, RASFs or MH7A cells were lysed in Lysis Buffer J, supplemented with protease inhibitors and centrifuged at 14,000 g for 3 min. After centrifugation, the cytoplasmic and nuclear fractions were collected from the supernatant and the pellet, respectively. Then, Buffer SK was used to extract RNA from the cytoplasmic and nuclear fraction. Subsequently, RT−qPCR was used to detect the expression of purified RNA.

### 4.10. Ribonuclease R (RNase R) Digestion

To verify the circCDKN2B−AS_006 characteristics, 3 μg RNA was incubated for 30 min at 37 °C with or without 3 U/μg RNase R (Epicentre Technologies, Madison, WI, USA). Then, the expression levels of circCDKN2B−AS_006 and the linear counterpart mRNA CDKN2B−AS_006 were determined by RT−qPCR and RT−PCR.

### 4.11. Actinomycin D Assay

RASFs or MH7A cells were seeded in a 6-well plate overnight and then treated with 2 mg/L actinomycin D (Sigma, Saint Louis, MO, USA) at the indicated time. The RT−qPCR assay was performed to assess the stability of circCDKN2B−AS_006 and CDKN2B−AS_006 after cells were harvested.

### 4.12. Sanger Sequencing

To further ascertain the back−splicing and junction sequence of circCDKN2B−AS_006, Sanger sequencing by Tsingke (Nanjing, China) was applied. The divergent primer (Invitrogen, Shanghai, China) was designed to confirm the back−splice junction of circCDKN2B−AS_006.

### 4.13. 5−Ethynyl−20−Deoxyuridine (EdU) Incorporation Assay

This assay was performed according to the method of a previous study [45]. To assess the proliferation viability of cells, the EdU assay was carried out with a BeyoClickTM EdU−555 detection kit (Beyotime, Shanghai, China). Transfected RASFs or MH7A cells were seeded in twelve-well plates and incubated with complete medium for 12 h. After incubation with 50 mM EdU for 6 h, the cells were fixed and stained for 30 min. The nucleic acid was stained with Hoechst 33342. All images were captured with a fluorescent microscope (Olympus Optical Co., Ltd., Tokyo, Japan).

### 4.14. Transwell Assay

This assay was performed according to the method of a previous study [45]. The migration and invasion abilities of RASFs or MH7A cells were assessed via transwell assays. Cells were seeded into the upper chamber, which was pre−treated with or without Matrigel (BD Biosciences, Franklin Lakes, NJ, USA), and each chamber was loaded in 100 μL serum-free culture medium and placed in 24−well tissue culture dishes. The lower chambers were filled with 600 μL DMEM containing 10% FBS. After 24 h of incubation, upper-chamber cells were removed and invaded cells were fixed and stained. All images were captured with a microscope (Olympus Optical Co., Ltd., Tokyo, Japan).

### 4.15. RNA−Binding Protein Immunoprecipitation (RIP) Assay

The EZ-Magna RIP RNA−binding protein immunoprecipitation kit 17−701 (Merck Millipore, Darmstadt, Germany) was used to conduct the RIP assay according to the manufacturer’s instructions. Briefly, RASFs or MH7A cells were washed with PBS and lysed in the RIP lysis buffer containing protease and ribonuclease inhibitors. The cell lysates were incubated with magnetic beads conjugated with Ago2 antibodies (Abcam, #ab156870) or IgG (Millipore, Billerica, MA, USA) at 4 °C overnight. Afterwards, beads were washed with a washing buffer and the complexes were incubated with Proteinase K for 30 min at 55 °C to remove the protein. Finally, the extracted purified RNAs were analyzed via RT−qPCR.

### 4.16. RNA Pull−Down Assay

GenePharma (Shanghai, China) designed and synthesized the biotinylated miR-1258 probes. The miR-1258 probe was incubated with C−1 magnetic beads (Life Technologies, Waltham, MA, USA) at 25 °C for 2 h to generate probe−coated beads. Afterwards, RASFs or MH7A cells were harvested and lysed, and the lysates were incubated with the miR−1258 probe or the negative control (NC) probes at 4 °C overnight. The RNA complexes combined with the beads were extracted using the RNeasy Mini Kit (QIAGEN). The abundances of circCDKN2B−AS_006 were evaluated via RT−qPCR. The probe sequences are listed in Supplementary Table S1.

### 4.17. Luciferase Reporter Gene Assay

Luciferase reporter plasmids were obtained from GenePharma (Shanghai, China). HEK−293T cells were seeded in 24−well plates and then co−transfected with circCDKN2B−AS_006/RUNX1 wild-type or mutant plasmids and miR−1258 mimics or NC mimics using Lipofectamine 2000. At 48 h after transfection, cells were lysed and the luciferase activities were measured using the luciferase reporter assay (Promega, Madison, WI, USA), according to the manufacturer’s instructions.

### 4.18. Bioinformatics Analysis

The sequence of circCDKN2B−AS_006 was obtained from the circBase database (http://www.circbase.org/; accessed on 15 February 2022). The structure of circCDKN2B−AS_006 was analyzed using circPrimer 1.2 software. The target miRNAs of circCDKN2B−AS_006 were predicted with the circular RNA interactome (https://circinteractome.nia.nih.gov; accessed on 15 February 2022). The potential target genes of miR−1258 were predicted using miRDB (http://mirdb.org/miRDB/; accessed on 15 February 2022) and miRwalk (http://mirwalk.umm.uni-heidelberg.de/; accessed on 15 February 2022).

Three gene expression profile datasets, GSE77298 (16 RA samples and 7 healthy controls), GSE1919 (5 RA samples and 5 healthy controls), and GSE55235 (10 RA samples and 10 healthy controls), were downloaded from the Gene Expression Omnibus (GEO) database (https://www.ncbi.nlm.nih.gov/geo; accessed on 19 March 2022). The series matrix and platform TXT files were downloaded from the GEO database. According to the annotation information in the platform, the probes were transformed into corresponding gene symbols. If a gene symbol was recorded with multiple probes, the average value was used as its expression level. The average expression of RUNX1 mRNA in the synovia of patients with RA and normal controls were compared.

Gene set enrichment analysis (GSEA) was conducted to explore the correlation between RUNX1 expression and RA−related gene set enrichment in GSE77298, GSE55235, and GSE55584 (http://software.broadinstitute.org/gsea/index.jsp; accessed on 6 May 2022).

Potential transcription factor RUNX1 binding sites were predicted using the known transcription factor binding site motifs from the TRANSFAC Predicted Transcription Factor Targets database. A Kyoto Encyclopedia of Genes and Genomes (KEGG) pathway analysis was performed using the clusterProfiler to identify the binding sites of RUNX1−enriched signaling pathways.

### 4.19. Animal Experiments

DBA/1 male mice (8 weeks, 20–24 g) were purchased from Beijing HFK Bioscience Co. (Beijing, China). All the animal experiments in this study were performed in strict accordance with the guidelines of the University Animal Care and Use Committee and were approved by the Animal Experimental Ethics Review Committee of Anhui Medical University (LLSC20210469).

After a week of adaptation, mice were randomly divided into 5 groups (10 mice/group) including the control group, model group, shcontrol group, shcircCDKN2B−AS_006 group, and MTX group. All mice except those in the control group were injected with complete Freund’s adjuvant (CFA) mixed with bovine collagen II (CII) (Chondrex Inc., Woodinville, WA, USA) and were given a booster injection of incomplete Freund’s adjuvant (IFA) mixed with CII on day 21 to establish the CIA mouse model. From the 28th day, mice in the shcontrol group and the shcircCDKN2B−AS_006 group were injected intra−articularly at the ankle with lentivirus stocks expressing shRNA for control or circCDKN2B−AS_006 at a concentration of 5 × 10^8^ virus particles per milliliter every 4 days; mice in the control group and the model group were injected intra−articularly at the ankle with PBS every 4 days; mice in the MTX group were intragastrically administered MTX at a 1 mg/kg dose every 3 days. The severity of arthritis was evaluated as previously described [46]. The mice were euthanized on Day 47 after the initial immunization. The ankle joints were collected and the soft tissues and muscles around the joints were removed and fixed with 4% paraformaldehyde solution for 48 h, decalcified in 10% ethylenediaminetetraacetic acid (EDTA) for 4 weeks, and embedded in paraffin. Subsequently, paraffin−embedded tissues were cut into 4 mm sections. For immunohistochemical staining, the experiment was performed using SP−9000 Histostain−Plus kits (Zsgb Bio, Beijing, China) according to the manufacturer’s protocols. Hematoxylin and eosin (H&E), toluidine blue O, and safranin O/fast green staining (Beyotime, Beijing, China) were performed according to the manufacturer’s protocols. All sections were scanned with a digital pathology slide scanner (3DHISTECH; The Digital Pathology Company, Budapest, Hungary). The average integrated optical density (IOD) of the immunohistochemical sections was calculated with Image−ProPlus software (Media Cybernetics, Silver Spring, MD, USA).

### 4.20. Statistical Analysis

The data were analyzed using SPSS (Statistical Package for the Social Sciences) version 17.0.1 (SPSS Inc., Chicago, IL, USA). A *p*-value < 0.05 was considered statistically significant, and the data are expressed as the mean ± standard error of the mean (SEM). Repeated-measures analysis of variance (ANOVA) followed by a least significant difference (LSD) test was performed to analyze the between-group effects on body weight and the polyarthritis index. Comparisons between the two groups were assessed via Student’s t−test. Comparisons between three or more groups were assessed via one−way ANOVA using an LSD post-hoc test.

## Data Availability

Not applicable.

## Supplementary Materials

The following supporting information can be downloaded at: https://www.mdpi.com/article/10.3390/ijms24065880/s1.
