# Supplementary material for: Circular RNA CircCDKN2B−AS_006 Promotes the Tumor-like Growth and Metastasis of Rheumatoid Arthritis Synovial Fibroblasts by Targeting the miR−1258/RUNX1 Axis
Source: Int J Mol Sci. 2023 Mar 20;24(6):5880. doi: 10.3390/ijms24065880 (PMC10051600; doi:10.3390/ijms24065880)
Supplement: Supplementary file 1 [file ijms-24-05880-s001.zip › Supplementary figure legends.pdf]

Supplementary figure legends

Figure. S1 CircCDKN2B-AS_006 promotes the proliferation, migration, and invasion of MH7A cells *in vitro*. **A**. The growth curves of cells were evaluated by CCK-8 assays after knocking down and overexpressing circCDKN2B-AS_006 in MH7A cells. **B** and **E**. EdU assays showing that knockdown of circCDKN2B-AS_006 inhibited the DNA synthesis of MH7A cells, while overexpression of circCDKN2B-AS_006 promoted DNA synthesis in MH7A cells. **C**-**D** and **F**. Transwell assays were performed to assess the migration and invasion abilities of MH7A cells. The samples were imaged at 100× magnification. The samples were imaged at 100× magnification. **$P < 0.01$.

Figure. S2 MiR-1258 inhibits the proliferation, migration, and invasion of RASFs *in vitro*. **A**. The growth curves of cells were evaluated by CCK-8 assays in RASFs after transfection with miR-1258 mimics or inhibitor. **B** and **E**. EdU assays showing that miR-1258 mimics inhibited the DNA synthesis, while miR-1258 inhibitor promoted DNA synthesis in RASFs. **C**-**D** and **F**. Transwell assays were performed to assess the migration and invasion abilities of RASFs. The samples were imaged at 100× magnification. **$P < 0.01$.

Figure. S3 MiR-1258 inhibits the proliferation, migration, and invasion of MH7A cells *in vitro*. **A**. The growth curves of cells were evaluated by CCK-8 assays in MH7A cells after transfection with miR-1258 mimics or inhibitor. **B** and **E**. EdU assays showing that miR-1258 mimics inhibited the DNA synthesis, while miR-1258 inhibitor promoted DNA synthesis in MH7A cells. **C**-**D** and **F**. Transwell assays were performed to assess the migration and invasion abilities of MH7A cells. The samples were imaged at 100× magnification. **$P < 0.01$.

Figure. S4 MiR-1258 reverses circCDKN2B-AS_006-promoted cell proliferation, migration, and invasion of MH7A cells. **A**-**B** and **E**. The cell proliferation were determined after transfection with sh-circCDKN2B-AS_006 and miR-1258 inhibitor

by CCK-8 (A) and EdU assays (B and E). **C**-**D** and **F**. The cell migration and invasion were determined after transfection with sh-circCDKN2B-AS_006 and miR-1258 inhibitor by transwell assays. **P < 0.01.

Figure. S5 CircCDKN2B-AS_006 promotes MH7A cells proliferation, migration and invasion via the miR-1258/RUNX1 axis. **A**-**B** and **E**. The cell proliferation were determined after transfection with sh-circCDKN2B-AS_006 and RUNX1 overexpression by CCK-8 (A) and EdU assays (B and E). **C**-**D** and **F**. The cell migration and invasion were determined after transfection with sh-circCDKN2B-AS_006 and RUNX1 overexpression by transwell assays. **P < 0.01.

Figure. S6 Bioinformatics analysis identified the potential relationship between RUNX1 with EMT process and Wnt signaling pathway in the synovium from RA patients. **A**. Relationship of ECM receptor interaction, adherens junction, and TGF-β signaling pathway with RUNX1 expression was shown using GSE77298. **B**. Relationship of focal adhesion, ECM receptor interaction, and Wnt signaling pathway with RUNX1 expression was shown using GSE55235. **C**. Relationship of ECM receptor interaction with RUNX1 expression was shown using GSE55584. **D**. Functional enrichment analysis of genes with RUNX1 transcription factor binding sites from the TRANSFAC Predicted Transcription Factor Targets database was assessed by KEGG pathway. **E**. Upset Venn diagram was used to identify the overlapped genes among the KEGG pathways related to EMT process, Wnt/β-catenin pathway, and cancer. **F**. Correlation analysis between RUNX1 and CTNNB1 expression in the synovium from RA patients in the gene expression microarray dataset (GSE55235).
