# Supplementary material for: Circular RNA CircCDKN2B−AS_006 Promotes the Tumor-like Growth and Metastasis of Rheumatoid Arthritis Synovial Fibroblasts by Targeting the miR−1258/RUNX1 Axis
Source: Int J Mol Sci. 2023 Mar 20;24(6):5880. doi: 10.3390/ijms24065880 (PMC10051600; doi:10.3390/ijms24065880)
Supplement: Supplementary file 1 [file ijms-24-05880-s001.zip › Supplementary table S1.pdf]

Table S1. The sequences of oligonucleotides and probes used in this study.

**miR-1258 mimics and inhibitors**	
mimics NC sense	UUCUCCGAACGUGUCACGUTT
mimics NC antisense	ACGUGACACGUUCGGAGAATT
miR-1258 mimics sense	AGUUAGGAUUAGGUCGUGGAA
miR-1258 mimics antisense	CCACGACCUAAUCCUAACUUU
inhibitor NC	CAGUACUUUUGUGUAGUCAA
miR-1258 inhibitor	UUCCACGACCUAAUCCUAACU
**FISH Probes**	
Cy3-circCDKN2B-AS_006 probe	TCTCATCAAAAGGGACACTCCAGTG
FAM-miR-1258 probe	TTCCACGACCTAATCCTAACT
**Biotinylated probes**	
Biotin-miR-1258 NC	TTCCACGACCTAATCCTAACT
Biotin-miR-1258	AGTTAGGATTAGGTCGTGGAA
**shRNAs**	
sh-circCDKN2B-AS NC (sh NC)	TTCTCCGAACGTGTCACGT
sh-circCDKN2B-AS	AGAAGACACTGGAGTGTCC
shRUNX1 NC	CCTAAGGTTAAGTCGCCCTCG
shRUNX1	GGCAGAAACTAGATGATCA
