# Supplementary material for: Circular RNA CircCDKN2B−AS_006 Promotes the Tumor-like Growth and Metastasis of Rheumatoid Arthritis Synovial Fibroblasts by Targeting the miR−1258/RUNX1 Axis
Source: Int J Mol Sci. 2023 Mar 20;24(6):5880. doi: 10.3390/ijms24065880 (PMC10051600; doi:10.3390/ijms24065880)
Supplement: Supplementary file 1 [file ijms-24-05880-s001.zip › Supplementary table S2.pdf]

Table S2. The sequences of primers used in this study.

Gene	Sequence (5'-3')
circCDKN2B-AS_006 Forward	TGGAGAAGACACTGGAGTGTC
circCDKN2B-AS_006 Reverse	TGAACCCTCTCTTTCCAAGA
CDKN2B-AS_006 Forward	TACAGGTGTGAGACACCACA
CDKN2B-AS_006 Reverse	TTCCCTGGTGTGTCCTTCAT
GAPDH Forward	GGAGCGAGATCCCTCCAAAAT
GAPDH Reverse	GGCTGTTGTCATACTTCTCATGG
divergent GAPDH Forward	GAAGGTGAAGGTCGAGTC
divergent GAPDH Reverse	GAAGATGGTGATGGGATTTC
RUNX1 Forward	CGAAGACATCGGCAGAAACT
RUNX1 Reverse	TAAAGGCAGTGGAGTGGTTCA
