# Supplementary figures and images for: Circular RNA CircCDKN2B−AS_006 Promotes the Tumor-like Growth and Metastasis of Rheumatoid Arthritis Synovial Fibroblasts by Targeting the miR−1258/RUNX1 Axis
Source: Int J Mol Sci. 2023 Mar 20;24(6):5880. doi: 10.3390/ijms24065880 (PMC10051600; doi:10.3390/ijms24065880)

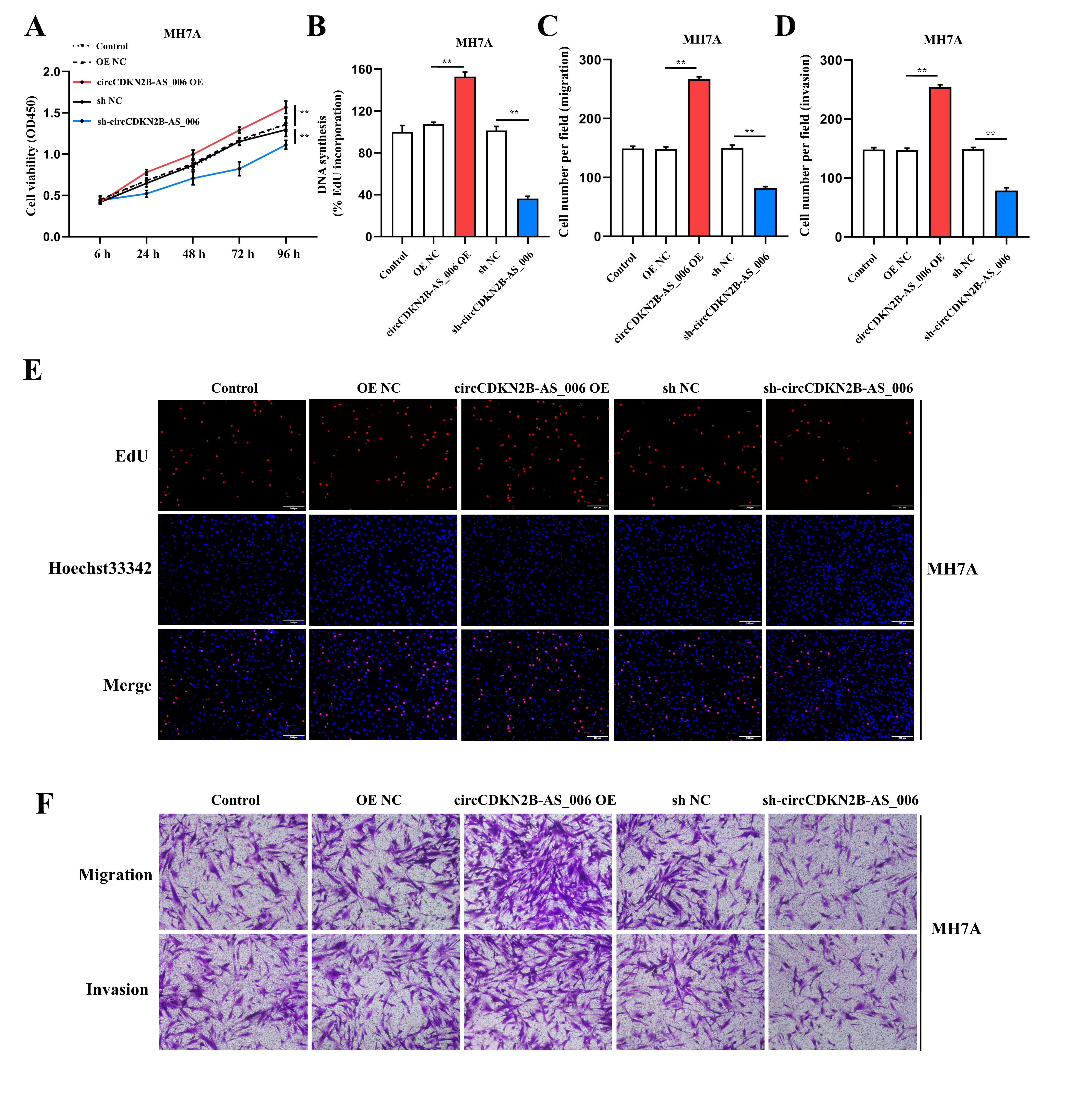

Supplement: Supplementary file 1 [file ijms-24-05880-s001.zip › Figure S1.tif]

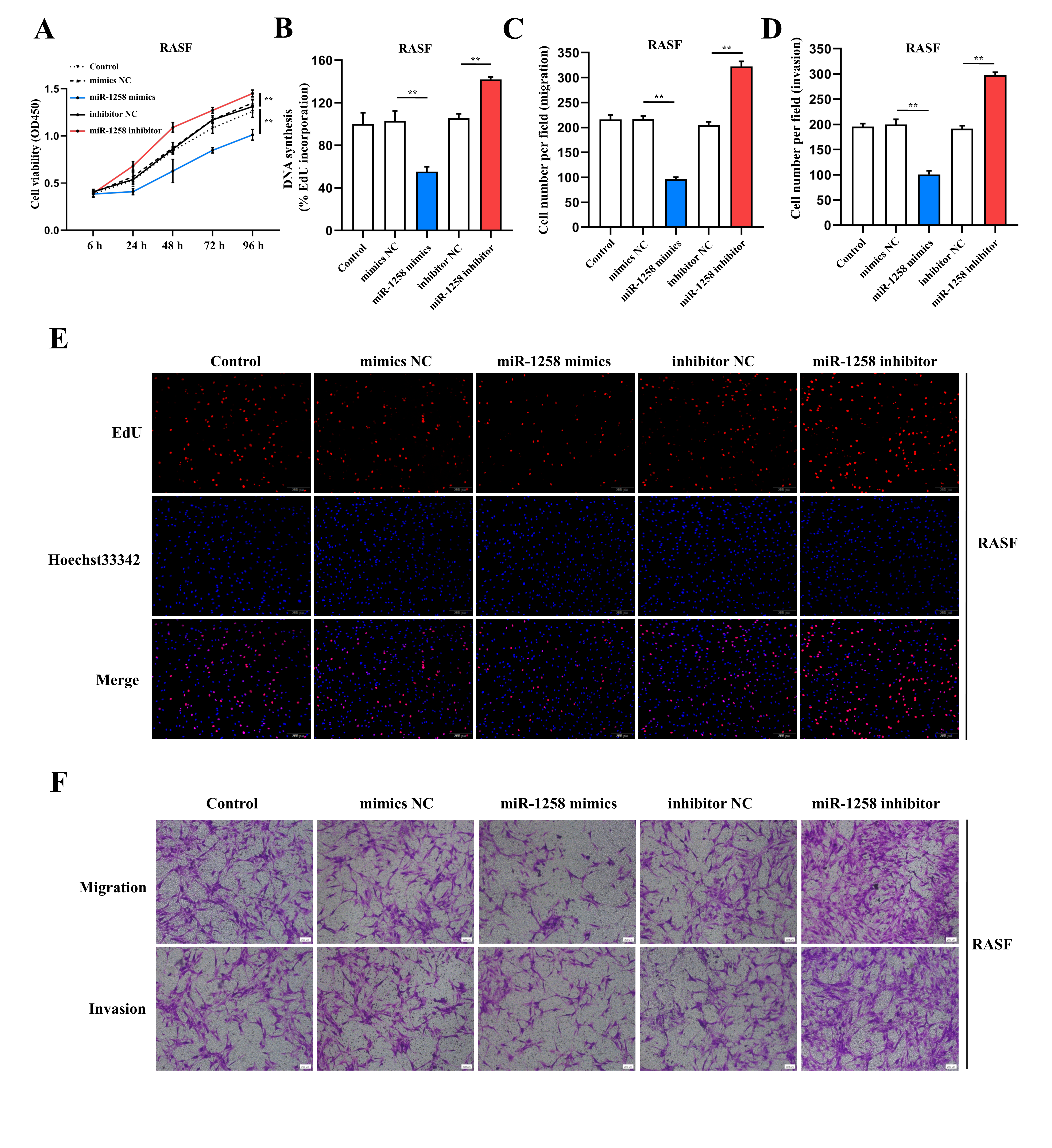

Supplement: Supplementary file 1 [file ijms-24-05880-s001.zip › Figure S2.tif]

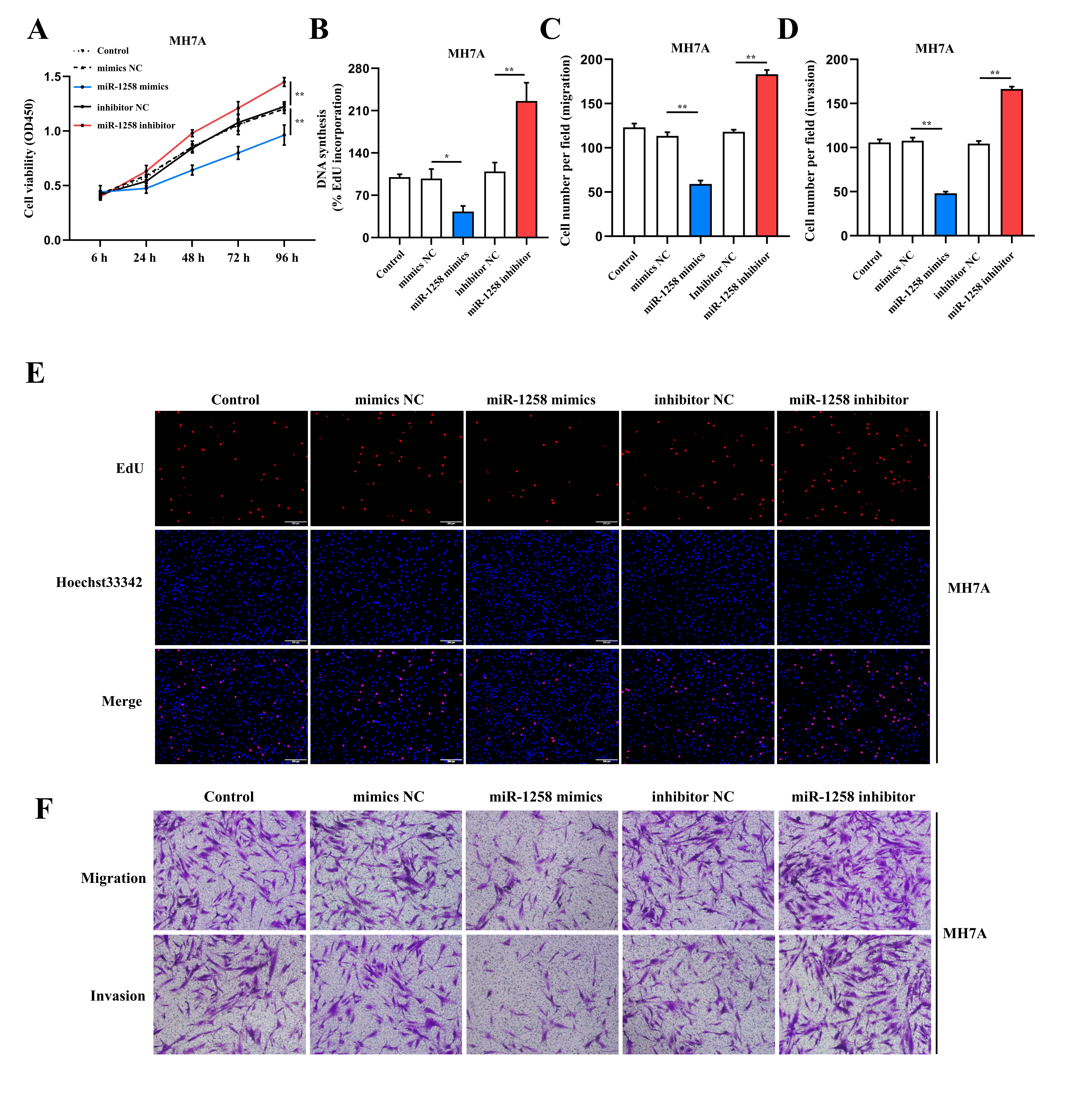

Supplement: Supplementary file 1 [file ijms-24-05880-s001.zip › Figure S3.tif]

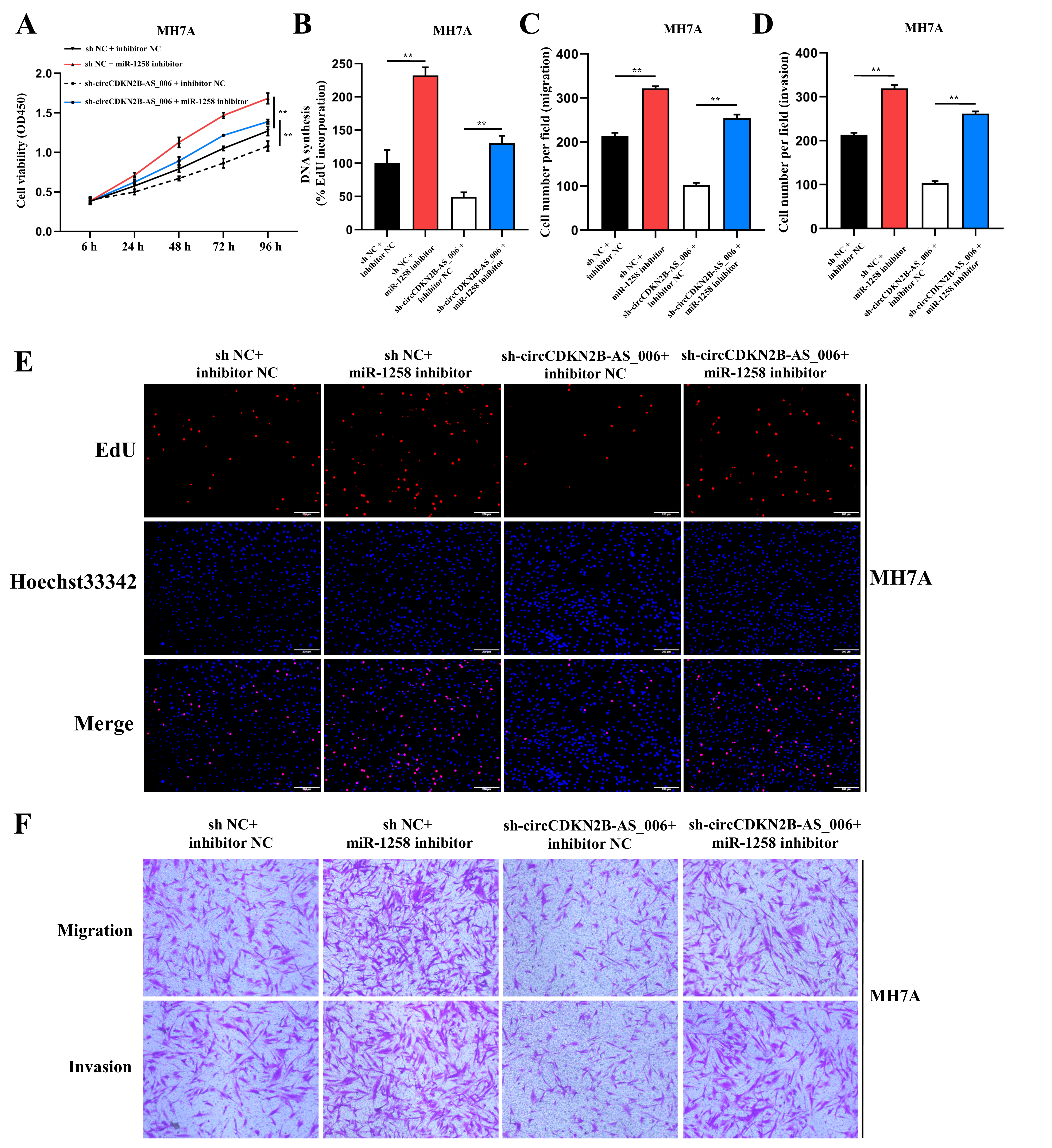

Supplement: Supplementary file 1 [file ijms-24-05880-s001.zip › Figure S4.tif]

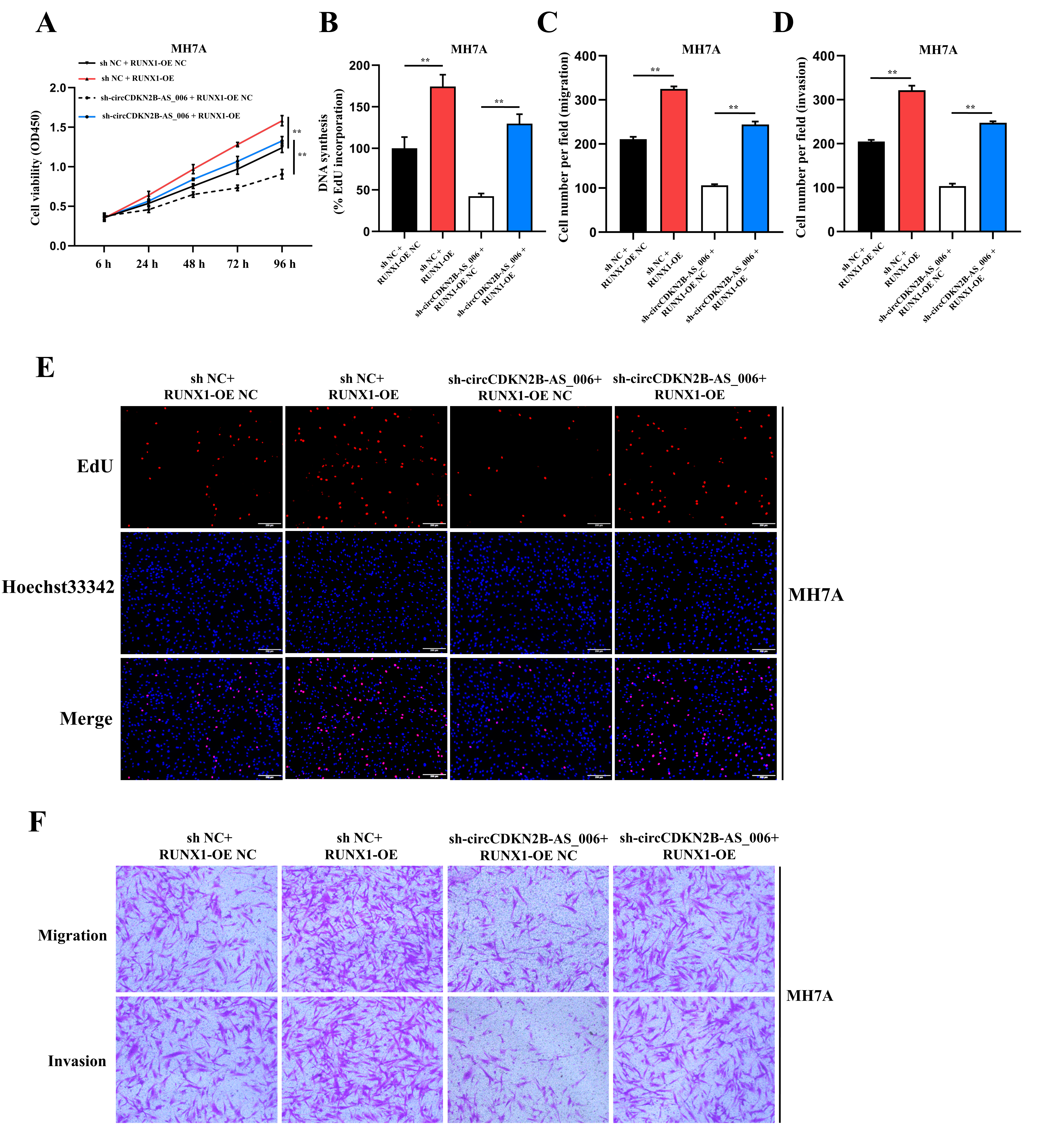

Supplement: Supplementary file 1 [file ijms-24-05880-s001.zip › Figure S5.tif]

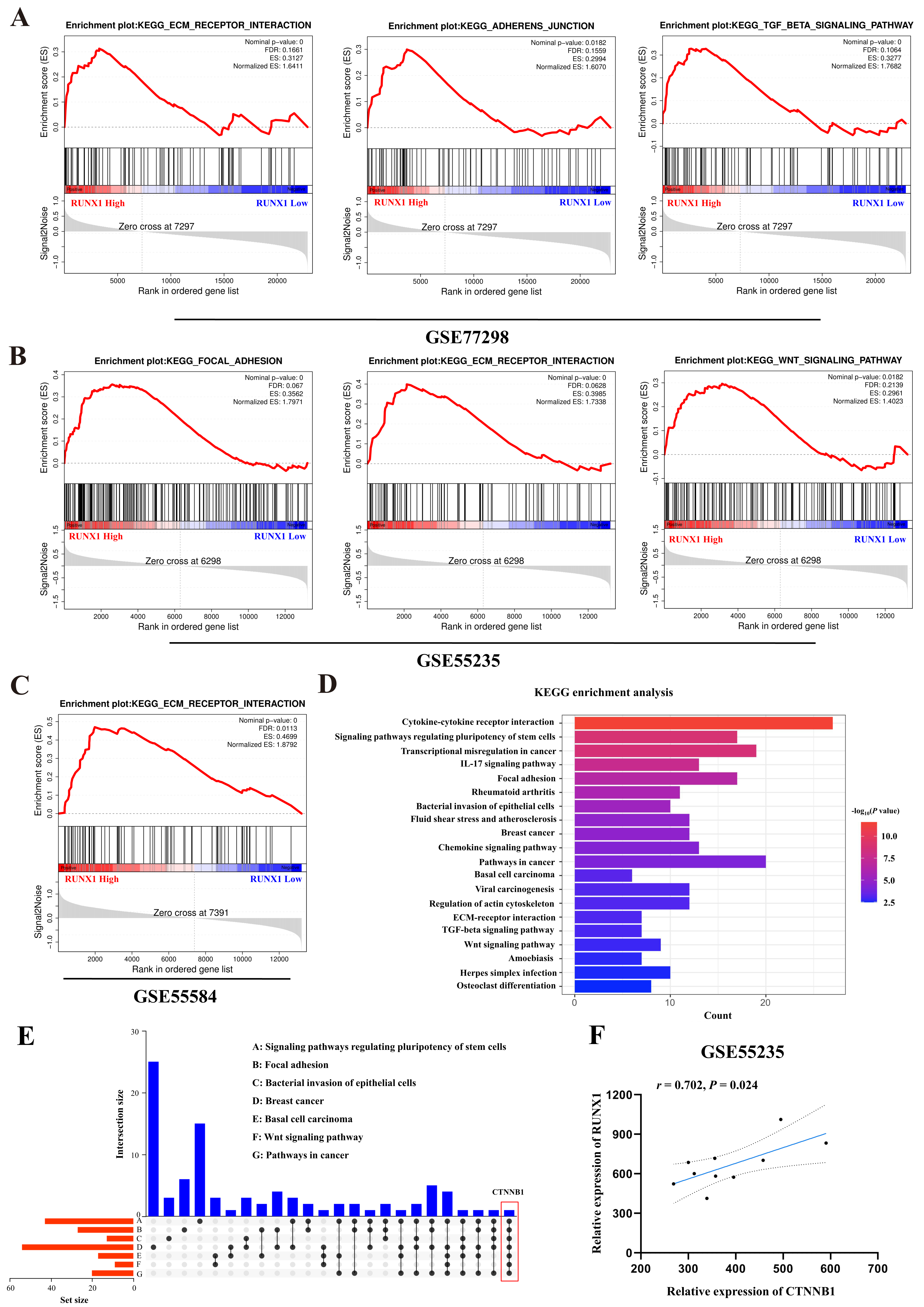

Supplement: Supplementary file 1 [file ijms-24-05880-s001.zip › Figure S6.tif]
